# Breeding system of diploid sexuals within the *Ranunculus auricomus* complex and its role in a geographical parthenogenesis scenario

**DOI:** 10.1002/ece3.7073

**Published:** 2020-12-03

**Authors:** Kevin Karbstein, Elisabeth Rahmsdorf, Salvatore Tomasello, Ladislav Hodač, Elvira Hörandl

**Affiliations:** ^1^ Department of Systematics, Biodiversity and Evolution of Plants (with Herbarium) Institute for Plant Sciences University of Göttingen Göttingen Germany; ^2^ Georg‐August University School of Science (GAUSS) University of Göttingen Göttingen Germany; ^3^ Institute of Biology Leipzig University Leipzig Germany

**Keywords:** experimental crossings, genetic distances, genome‐wide heterozygosity, outcrossing, RADseq, *Ranunculus auricomus*, selfing

## Abstract

The larger distribution area of asexuals compared with their sexual relatives in geographical parthenogenesis (GP) scenarios has been widely attributed to the advantages of uniparental reproduction and polyploidy. However, potential disadvantages of sexuals due to their breeding system have received little attention so far. Here, we study the breeding system of five narrowly distributed sexual lineages of *Ranunculus notabilis* s.l. (*R. auricomus* complex) and its effects on outcrossing, inbreeding, female fitness, and heterozygosity. We performed selfing and intra‐ and interlineage crossings by bagging 481 flowers (59 garden individuals) followed by germination experiments. We compared seed set and germination rates, and related them to genetic distance and genome‐wide heterozygosity (thousands of RADseq loci). Selfings (2.5%) unveiled a significantly lower seed set compared with intra‐ (69.0%) and interlineage crossings (69.5%). Seed set of intra‐ (65%) compared to interpopulation crossings (78%) was significantly lower. In contrast, all treatments showed comparable germination rates (32%–43%). Generalized linear regressions between seed set and genetic distance revealed positive relationships in general and between lineages, and a negative one within lineages. Seed set was the main decisive factor for female fitness. Germination rates were not related to genetic distance at any level, but were positively associated with heterozygosity in interlineage crossings. Experiments confirmed full crossability and predominant outcrossing among sexual *R. notabilis* s.l. lineages. However, up to 5% (outliers 15%–31%) of seeds were formed by selfing, probably due to semi‐self‐compatibility in a multi‐locus gametophytic SI system. Less seed set in intrapopulation crossings, and higher seed set and germination rates from crossings of genetically more distant and heterozygous lineages (interlineage) indicate negative inbreeding and positive outbreeding effects. In GP scenarios, sexual species with small and/or isolated populations can suffer from decreased female fitness due to their breeding system. This factor, among others, probably limits range expansion of sexuals.

## Graphical abstract

Potential disadvantages of sexual plant species due to their breeding system have received little attention in geographical parthenogenesis (GP) scenarios so far. We compared seed set and germination rates of five sexual lineages of *Ranunculus notabilis* s.l (*R. auricomus* complex) and related them to genetic distance and genome‐wide heterozygosity. Results suggest that inbreeding depression particularly affects intrapopulation (intralineage) crossings, and that positive outbreeding effects particularly influence interlineage crossings. In GP scenarios, sexual species with small and/or isolated populations can suffer from decreased female fitness due to their breeding system.

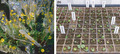

## 1 INTRODUCTION

Geographical parthenogenesis (GP) describes the phenomenon by which asexual taxa have larger distribution areas than their sexual relatives (Hörandl, 2006; Kearney, 2005; Vandel, 1928). Several factors may contribute to the pattern, whereby most hypotheses focus on the potential advantages of asexual taxa. In general, uniparental reproduction allows for a faster establishment of populations, and hence more efficient colonization of devastated areas (Hörandl, 2006). Reproductive assurance in the case of uniparental reproduction is a major advantage for asexuals in GP scenarios (e.g., Lo et al., 2013). Additionally or alternatively, positive side effects of polyploidy of asexual lineages may allow for niche shifts towards colder or more extreme climatic conditions (Bierzychudek, 1985; Lo et al., 2013; Paule et al., 2018). The success of asexuals can be also related to differential niche dynamics of clonal lineages versus sexual species (general purpose versus niche‐specific genotypes; Vrijenhoek & Parker, 2009).

So far little attention has been paid to the potential disadvantages of sexual species in GP scenarios. Sexuality in plants and animals is by far the most abundant and widespread mode of reproduction, and benefits from short‐term advantages of recombination (Burt, 2000; Maynard‐Smith, 1978). In most cases of GP scenarios, however, the sexual relatives of asexual complexes occur in relatively small distribution areas that are quite often interpreted as relict areas in glacial refugia during the Pleistocene (Bierzychudek, 1985; Cosendai & Hörandl, 2010; Hörandl et al., 2008; Lo et al., 2009). It is still enigmatic why sexual populations fail to expand their range in postglacial recolonization scenarios despite the ample availability of suitable habitats (e.g., Kirchheimer et al., 2018; Tomasello et al., 2020). Although differential niche dynamics, the occurrence of apomictic conspecifics, and ecological preferences can explain a geographical separation of sexual and asexual lineages in some cases (Karunarathne et al., 2018; Kirchheimer et al., 2018; Nardi et al., 2020), others do not confirm a scenario of niche separation (Mau et al., 2015). Sexual species are otherwise (without asexual congeners) also the most abundant colonizers in extremely high altitudes and latitudes (Asker & Jerling, 1992; Brožová et al., 2019; Hörandl et al., 2011). It remains an open question why sexual species are unsuccessful in GP scenarios.

In flowering plants, the diversity of breeding systems in sexual species is an important factor for these considerations. Most angiosperms are hermaphroditic and hence, self‐fertilization is a frequent option for reproduction. Selfing provides reproductive assurance even for isolated single plants or populations (Renner, 2014; Richards, 1997). Indeed, self‐fertility has been recognized as a general short‐term advantage for colonization and range expansion, when mating partners and pollinators are rare (Baker's law, Baker, 1967; Pannell, 2015). However, sexual selfing has the disadvantage of a rapid loss of heterozygosity over generations, which can cause inbreeding depression (Charlesworth & Charlesworth, 1987; Goldberg et al., 2010; Richards, 1997; Freeland et al., 2011). In contrast, asexual selfing in apomicts fixes the existing heterozygosity over generations due to the lack of meiosis and recombination (Brochmann et al., 2004; Hörandl, 2006). Most members of sexual‐apomictic complexes are long‐lived perennials (Asker & Jerling, 1992), for which the effects of inbreeding are expected to be more severe than in annuals. Purging effects of the mutational load from one generation to the next (Barrett & Charlesworth, 1991; Crnokrak & Barrett, 2002) are faster and more efficient in obligate selfing annuals than in perennials (Richards, 1997). Beside selfing, biparental inbreeding in small and isolated outcrossing populations could have also negative effects on fitness as has been suggested for example by experiments in *Hieracium* (Pinc et al., 2020). Haag and Ebert (2004) hypothesized that asexual colonizers in marginal populations would suffer less from genetic bottlenecks and subsequent drift than sexual populations. Fitness of sexuals would be reduced in such small colonizer populations due to inbreeding depression. Comprehensive population genetic studies on GP scenarios, however, either did not observe the respective genetic pattern (e.g., Cosendai et al., 2013) or found the colonization history and decline of genetic diversity in marginal sexual populations as less relevant for the GP pattern (e.g., Nardi et al., 2020). On the contrary, apomictic lineages can benefit from uniparental reproduction either via pollen‐independent (autonomous) apomixis (Mráz et al., 2018) or via pseudogamy and self‐compatibility (Hörandl, 2010) without negative effects of inbreeding. However, little is known about the effects of breeding systems, inbreeding, and fitness of sexual species in geographical parthenogenesis scenarios.

Most sexual progenitors of apomictic plants are reported to be self‐incompatible (Alonso‐Marcos et al., 2018; Cosendai et al., 2013; Hörandl, 2010; Vašková & Kolarčik, 2019). Hence, they are trapped in a twofold dilemma: as outcrossers, they do need conspecific mating partners and pollen vectors, which is problematic in small and isolated populations. Followingly, the founding of populations from single or few individuals is hampered after long‐distance dispersal (Baker' law; Pannell, 2015). However, also dispersal and gene flow with other, more adjacent conspecific populations will remain limited, if lineages are distributed disjunctly in refugial areas. Inbreeding, loss of genetic diversity via drift, loss of heterozygosity, and eventually inbreeding depression are to be expected in such small and isolated populations. On the one hand, facultative self‐fertilization would severe the process of loss of heterozygosity in perennial populations. On the other hand, occasional selfing could aid range expansion of sexual lineages and foster gene flow with other adjacent populations or lineages of the species. Such intercrossing of slightly diverged, but still cross‐compatible lineages might counteract the negative effects of inbreeding and preserve the genetic diversity of a species. However, effects of inbreeding, rare selfing, or intercrossing on genetic diversity of sexual lineages are poorly explored in the context of geographical parthenogenesis.

**Figure 1:**
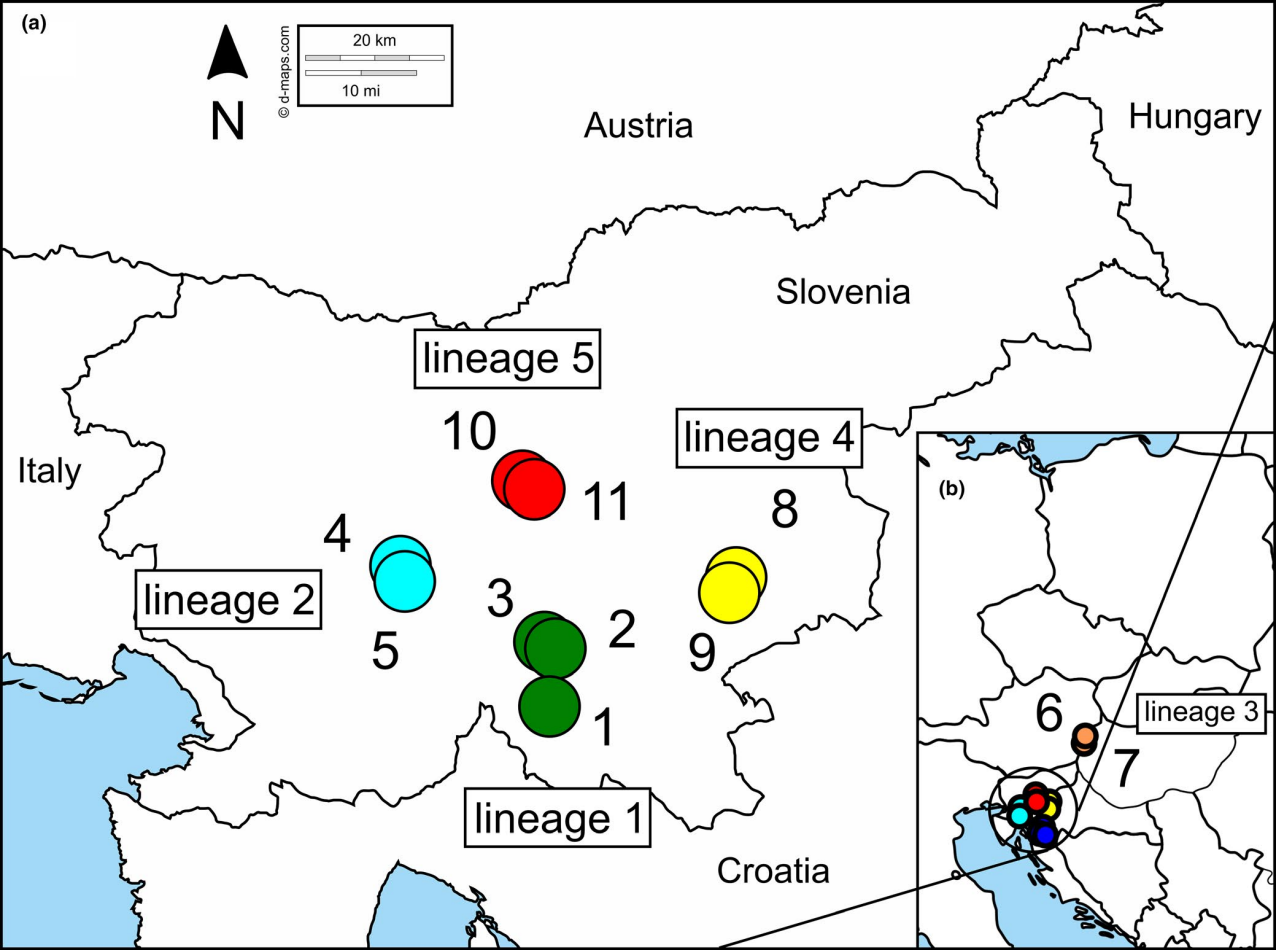
(a) Study locations of sexual *R. notabilis* s.l. populations in Slovenia (see also Karbstein et al., 2020), and (b) all study locations in a European view. Locations are indicated by circles with different numbers corresponding to Map IDs in Table 1. We downloaded the original map from https://d‐maps.com/

We focus here on *R. notabilis* s.l., a sexual species of the Eurasian *Ranunculus auricomus* complex, as a model system for studying the breeding system and the effects of genetic differentiation and genetic diversity (heterozygosity) among individuals/lineages on reproductive fitness. The *R. auricomus* complex shows a typical pattern of geographical parthenogenesis, with five allopatric sexual species in Southern to Central Europe (Karbstein et al., 2020; Tomasello et al., 2020), and a huge diversity of apomictic lineages occupying temperate to arctic Europe, Western Siberia, and Greenland (Hörandl, 2009; K. Karbstein, S. Tomasello, L. Hodač, M. Daubert, E. Lorberg, & E. Hörandl, unpublished data). The sexual species are restricted to very small and/or disjunct distribution areas, and are sometimes confined to single populations (Dunkel et al., 2018; Karbstein et al., 2020). Biogeographical analyses revealed an allopatric speciation process from the mid‐Pleistocene onwards, but could not answer the question why these plants of forest understory or meadow habitats failed to expand their range after postglacial reforestation of temperate Europe (Tomasello et al., 2020). Pollination experiments revealed self‐incompatibility of sexual taxa, whereby mostly samples of the temperate‐montane and Central European species *R. cassubicifolius* s.l. were included in this study (Hörandl, 2008). Tetraploid and hexaploid (Hörandl, 2008) apomicts were found to be self‐fertile. The seed set of sexual taxa was significantly higher than that of apomictic ones, while germination rates were not different between reproduction modes and ploidy levels (Hörandl, 2008). However, in this earlier study, very few samples of the other main sexual progenitor species in Central Europe, *R. notabilis*, could be included because only a few and very small populations were known from Southeastern Austria. Population genetic studies using isoenzymes suggested inbreeding in some of these small populations (Hörandl et al., 2000). However, population genetic studies applying more efficient DNA markers are missing so far. Recently, several local sexual lineages in Slovenia were detected (Dunkel et al., 2018), but these were also classified within *R. notabilis* s.l. according to comprehensive phylogenomic and geometric morphometric studies (Karbstein et al., 2020; Tomasello et al., 2020). The species *R. notabilis* s.l. is structured into six locally distributed lineages within the Illyrian region (incl. Southeastern Austria; Figure 1), but lineage ranges are partially overlapping (Dunkel et al., 2018; Tomasello et al., 2020). *Ranunculus notabilis* s.l. represents an established model system appropriate for studying the breeding system, effects of crossability within and between local lineages, and reproductive fitness measures.

By using experimental crossings and analyses of key genetic measures, we aim at clarifying the role of breeding systems of sexual plant species in GP scenarios. We address the following questions: (a) Are lineages within *R. notabilis* s.l. self‐incompatible, or does facultative self‐fertility occur? (b) Are these lineages fully cross‐compatible, as expected within a species? (c) Do interlineage crossings differ in their seed set from intralineage ones (in comparison with other sexual species of the *R. auricomus* complex to exclude general crossing barriers/inbreeding)? (d) Do germination rates differ in inter‐ and intralineage crossings? (e) How do genetic distance and genome‐wide heterozygosity differ among lineages? (f) How do these measures relate to reproductive fitness?

## 2 MATERIALS AND METHODS

### 2.1 Study locations and population sampling

We included five lineages of *R. notabilis* s.l. in the present study, covering the whole range of this Illyrian species. Four of them were formerly described by Dunkel et al. (2018) as distinct species, but were lumped to *R. notabilis* s.l. by Karbstein et al. (2020) due to close phylogenetic relationships and similar morphology in leaf and receptacle traits (lineage 1 “*R. austroslovenicus*,” lineage 2 “*R. mediocompositus*,” lineage 3 *R. notabilis* s.str. (Austria), lineage 4 “*R. peracris*,” and lineage 5 “*R. subcarniolicus*”). Up to 16 individuals per wild population and one to three populations per lineage were sampled in Slovenia and Austria from 2011 to 2018 (Table 1, Figure 1). We took living plants and cultivated them in the Old Botanical Garden at the University of Göttingen (controlled environmental conditions for solar radiation and water supply) for crossing experiments. As described in Karbstein et al. (2020), we collected herbarium specimens (deposited in GOET), and recorded altitude, GPS coordinates, and habitat characteristics of each population (Table 1, Figure 1). All lineages prefer humid and semi‐shaded habitats in the submontane forest zone (see Hörandl & Gutermann, 1998; Dunkel et al., 2018). Therefore, we assume that seed formation and germination under garden conditions, where we also applied semi‐shading, were not affected by different original habitat preferences. We used silica gel dried fresh leaves for further genetic laboratory work. Diploid ploidy level and sexual reproduction mode were previously confirmed by Karbstein et al. (2020).

**Table 1:** Locations of sexual diploid lineages of *R. notabilis* s.l. in the Illyrian region (see also Karbstein et al., 2020). Population ID with map ID (see Figure 1), species, lineage number, number of garden individuals involved in crossings (*N*_Cross_), number of RADseq samples (*N*_RAD_), locality by country, collection date of population sampling, altitude (in meter above sea level, m.a.s.l.), latitude (N, decimal), longitude (E, decimal), habitat, collector, and herbarium voucher specimens. Former and no longer accepted sexual species names after Dunkel et al. (2018) are described in Materials and methods or can be assessed via population IDs in Dunkel et al. (2018) and Karbstein et al. (2020). Populations Du‐30441, Du‐30442, EH10137, and Du‐33266 were not involved in crossings. Herbarium voucher codes are given in Karbstein et al. (2020)

Pop ID	Map ID	Species	Lineage	*N*_CROSS_	*N*_RAD_	Locality	Collection date	Altitude	Latitude (N)	Longitude (E)	Habitat	Collector	Herbarium Voucher
*Du‐30441*	(1)	*R. notabilis* s.l.	1	–	1	Slovenia	23.04.2013	260	45.462527	14.817019	Shrubbery, forest edge	F.G.Dunkel	LJU, B, M
*Du‐30442*	(2)	*R. notabilis* s.l.	1	–	1	Slovenia	23.04.2013	470	45.684671	14.810336	Forest	F.G.Dunkel	M
*LH012*	(3)	*R. notabilis* s.l.	1	8	2	Slovenia	03.05.2017	469	45.677445	14.82614	Forest	L.Hodač, K.Spitzer	GOET
*LH014*	(4)	*R. notabilis* s.l.	2	8	2	Slovenia	03.05.2017	434	45.84954	14.25946	Humid meadow	L.Hodač, K.Spitzer	GOET
*LH015*	(5)	*R. notabilis* s.l.	2	7	2	Slovenia	03.05.2017	436	45.848988	14.257088	Marshy meadow	L.Hodač, K.Spitzer	GOET
*EH10137*	(6)	*R. notabilis* s.l.	3	–	2	Austria	08.05.2011	220	47.047778	16.4325	Forest edge, meadow	E.Hörandl, S.Hörandl, F.Hadacek	GOET
*LH028*	(7)	*R. notabilis* s.l.	3	4	1	Austria	13.04.2018	227	47.053221	16.435162	Forest edge, meadow	L.Hodač, K.Spitzer	GOET
*LH010*	(8)	*R. notabilis* s.l.	4	12	2	Slovenia	02.05.2017	151	45.89075	15.370933	Forest edge	L.Hodač, K.Spitzer	GOET
*LH011*	(9)	*R. notabilis* s.l.	4	11	2	Slovenia	02.05.2017	148	45.880803	15.336522	Forest edge	L.Hodač, K.Spitzer	GOET
*Du‐33266*	(10)	*R. notabilis* s.l.	5	–	1	Slovenia	26.04.2016	325	45.944056	14.653083	Forest edge	F.G.Dunkel	M
*LH013*	(11)	*R. notabilis* s.l.	5	8	2	Slovenia	03.05.2017	324	45.945453	14.651618	Humid meadow	L.Hodač, K.Spitzer	GOET

### 2.2 Crossing and germination experiments

**Figure 2:**
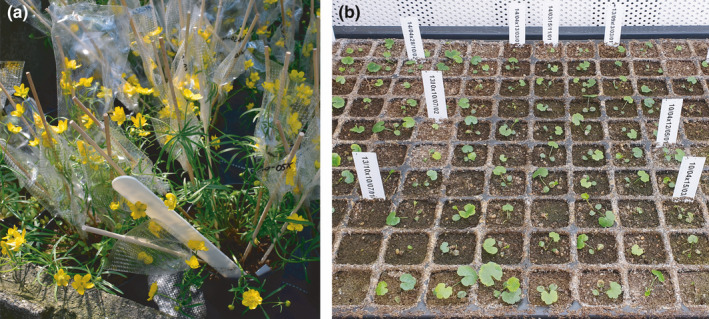
(a) Crossings experiments were conducted in the Old Botanical Garden at the University of Göttingen. *Ranunculus notabilis* s.l. individuals were bagged with porous plastics bags (bags sealed with tape and labeled) and cultivated under controlled environmental conditions. Viable seeds were sowed out and cultivated in (b) climate chambers for germination rate experiments at the University of Göttingen. Labels separate different crossing IDs

We conducted manual crossings in 2019 (after at least one year of plant cultivation under the same garden conditions) following two different treatments: among individuals of a lineage including intra‐ and interpopulation crossings (“intralineage”) and among individuals of different lineages (“interlineage”; see also Hörandl, 2008). Individual crossing pairs are shown in the Excel file “crossings_2019.xlsx” deposited on Dryad data repository. We isolated single flowers (not emasculated) in the bud stage using porous plastic bags (Baumann Saatzuchtbedarf GmbH, Waldenburg, Germany) to avoid undesired cross‐pollination. Plastic bags were voluminous enough not to be touched by androecium and gynoecium. This avoids accidental pollination by insects “walking” on the bag surfaces (see Figure 2a). Isolated flowers were not emasculated in intra‐ and interlineage crossings because this procedure injures the flowers severely and makes them susceptible to fungal infections, often resulting in flower abortion (pers. experience of the senior author). Moreover, sexual *R. auricomus* species were observed to be absolutely self‐incompatible so far (Hörandl, 2008). When individuals were met in the flowering stage and pollen grains were visible, we conducted the above‐mentioned cross‐pollination treatments. Cross‐pollination of flowers was repeated at least once to ensure successful pollination. Due to this crossing procedure, we ensured that intra‐ or interlineage pollen was the dominant part of crossings. We regard self‐fertilization via mentor effects as unlikely (see Discussion). Additionally, we isolated single flowers before anthesis with porous plastic bags to test for self‐incompatibility (“selfing”; see also Hörandl, 2008). We included 8, 15, 4, 23, and 8 individuals for lineage 1, 2, 3, 4, and 5, respectively (see Table 1). In total, we bagged 481 flowers and 59 garden individuals. Afterward, bags were sealed with tape to prevent seed loss during harvesting (see Figure 2a).

We harvested ripened achenes from May to June 2019. Per bag (one flower per bag), achenes were categorized into viable versus aborted achenes and counted separately. Viable achenes were recognized by color (brown to dark brown), endosperm development (hard and full achene bodies), size (larger), and removability from the receptaculum (easily falling off; see also Hörandl, 2008). Since *Ranunculus* has single‐seeded achenes, the proportion of viable achenes equals proportions of viable seeds from all seeds (= seed set). In 2020, we added the seed set of three additional individuals of lineage 3 to increase the sample size.

To preserve germinability for germination experiments, we stored seeds in plastic boxes at 7°C (fridge). Cool and dark storage should simulate autumn/winter conditions to overcome seed dormancy (see Lohwasser, 2001 and Vandelook, 2009 for *R. auricomus*). We sowed seeds on the 19th of September 2019 and stored pots under similar garden conditions (garden beds were protected against direct rain and sunlight). To prevent frost damage on young seedlings, we transferred pots into climate growth chambers and raised plants under conditions of 10 hr photoperiod and 12°C air temperature (see Figure 2b). Pricking out of plants started on the 23rd of January 2020 and was finished on the 27th of March 2020. Within this period, we counted germinated seeds per pot.

### 2.3 Laboratory work—DNA extraction and RADseq

We extracted the DNA of 18 *R. notabilis* s.l. individuals (representing three to four samples per lineage) as described in Karbstein et al. (2020). We prolonged sample incubation in lysis buffer to one hour for more DNA yield. The DNA concentration was checked using Qubit fluorometer and Qubit dsDNA BR Assay Kit (ThermoFisher Scientific, Waltham, USA). We targeted a final DNA solution of 30 ng/μl in a volume of 55 μl and visually checked DNA quality by gel electrophoresis. Samples were analyzed by Floragenex Inc. (Portland, USA). The generation of RAD libraries was based on the protocol of Baird et al. (2008), and the enzyme *PstI* was used for digestion. Sequencing was performed on an Illumina® HiSeq 4,000 platform to produce 100 bp single‐end reads at the University of Oregon Genomics Core Facility. The quality of raw reads was checked with FastQC vers. 0.11.8 (Andrews, 2010).

### 2.4 Data analyses

All statistical analyses were performed with R vers. 4.0.0 (R Core Team, 2020). We calculated seed set per bag, and germination rates as the ratio of germinated seeds to viable seeds. We averaged seed set and germination rate per crossing ID (e.g., LH010‐4 × LH028‐10). For statistical analyses, our final sample size was *N* = 284 for seed set and *N* = 260 for germination rates.

To examine differences between seed set or germination rates and different treatments (selfing, intralineage (intra‐ and interpopulation), and interlineage) of different *R. notabilis* s.l. lineages, we performed Kruskal–Wallis tests (for non‐normal distribution of proportional data) to check for significant group differences. If results were significant, we calculated pairwise Wilcoxon rank‐sum tests with Holm correction to assess pairwise group differences.

To evaluate seed set and germination rates in relation to genetic distance, we performed de novo assembly of RADseq loci and parameter optimization in IPYRAD vers. 0.9.14 (Eaton, 2014; Eaton & Overcast, 2020) on the GWDG HPC Cluster (Göttingen, Germany). We created a subset of 18 sexual *R. notabilis* s.l. samples out of the total 45 sexual *R. auricomus* ones used by Karbstein et al. (2020; see Table 1). Raw read demultiplexing, removal of adapter sequences and restriction overhang, further quality filtering, and selection of in‐sample clustering threshold (ISCT = 95%) and between sample clustering threshold (BSCT = 92%) exactly followed Karbstein et al. (2020; see also Paris et al., 2017; Pätzold et al., 2019). We selected the “min30” instead of the “min10” dataset, that is, a minimum of 30% available samples per locus (5 out of 18 samples), avoiding too high amounts of missing data in the final assembly. Analysis yielded 41,580 filtered loci, ranging from 13,873 to 27,457 loci/individual (mean 19,691 loci/individual by 52% missing sites in the final SNP matrix). In order to estimate the genetic distance among *R. notabilis* s.l. lineages, we computed a network analysis based on the IPYRAD *.u.snps output file (unlinked SNPs, one SNP per locus; converted into a *.nex file) and the Neighbor‐Net algorithm using the program SPLITSTREE vers. 4.14.6 (Huson & Bryant, 2006). We specified a general time‐reversible (GTR) model with estimated site frequencies and maximum likelihood (equal rates of site variation, default rate matrix; see also Karbstein et al., 2020). Genetic distances were exported from SPLITSTREE, and (mean) values were used in further data analyses.

To evaluate seed set and germination rates in relation to heterozygosity, we used genome‐wide heterozygosity values based on the RADseq dataset of the same 18 samples already calculated by K. Karbstein, S. Tomasello, L. Hodač, M. Daubert, E. Lorberg, & E. Hörandl (unpublished data; final assembly “min50”). Raw values, mean values per crossing ID, and mean values per lineage (e.g., mean of lineage 1 and 2) were used in further data analyses.

We performed the Kruskal–Wallis test to check for significantly different genetic distances among lineage combinations (e.g., lineage 1—lineage 1 (intralineage), lineage 1—lineage 2 (interlineage)). The same tests were also conducted to assess significant differences in heterozygosity among other sexual diploid *R. auricomus* species (heterozygosity values were also taken from K. Karbstein, S. Tomasello, L. Hodač, M. Daubert, E. Lorberg, & E. Hörandl, unpublished data; final assembly “min50”), and among sexual diploid *R. notabilis* s.l. lineages.

To evaluate relationships between seed set or germination rates and mean genetic distances (mean between parental lineage pairs; see Figure S8 for simple distances) or mean heterozygosity (mean between parental mother lineage and pollen donor lineage) of parental lineages, we performed simple quasibinomial generalized linear regression models (GLMs). We used mean values of parental lineages because no full correspondence between crossed and sequenced individuals was available (see also Excel file ‘crossings_2019.xlsx’ in Dryad data repository). Moreover, genetic cohesion in sexual (meta‐)populations (see e.g., Hörandl et al., 2001; Freeland et al., 2011), and the fact that lineages are slightly genetically differentiated (i.e., have some own genetic features, see also ML tree in Karbstein et al., 2020), also justify the use of lineage means in our regressions. Finally, results were plotted, and regression curves were drawn according to GLM results.

To study isolation‐by‐distance (IBD) among individuals of *R. notabilis* s.l. lineages, we first calculated pairwise geographic distances among locations applying the Vincenty method implemented in the R package “geosphere” vers. 1.5–10 (Hijmans et al., 2019). Second, we performed a Mantel test (Spearman; 9,999 permutations) between genetic (previously used) and geographical distances using the R package “ape” vers. 5.3 (Paradis et al., 2019). We plotted data points and the regression line.

## 3 RESULTS

**Figure 3:**
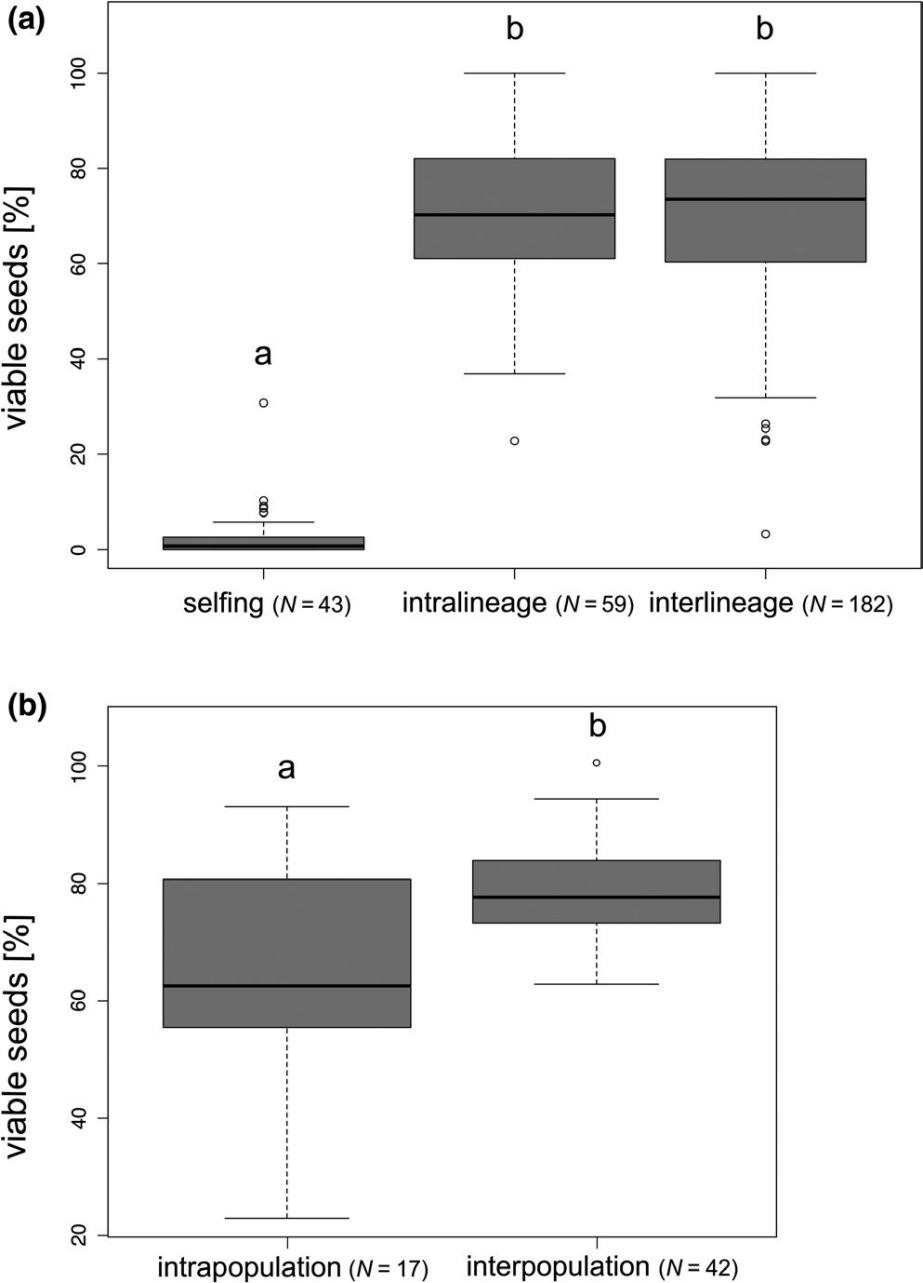
Boxplots showing (a) seed set (percent of viable seeds) of different treatments (selfing, intra‐, and interlineage) and (b) seed set of inter‐ and intrapopulation crossings (intralineage). Letters above boxplots indicate significant/nonsignificant differences between groups. *N* = sample size (number of crossings/crossing IDs used to determine seed set)

We observed significant differences between seed set and different treatments (Chi‐squared = 108.33, *df* = 2, *p* < .001; Figure 3a). Selfings revealed significant lower (mean 2.47%, *p* < .001) seed set compared with intra‐ and interlineage crossings (means 68.97% and 69.49%; Table 2, Figure 3a). Inter‐ and intralineage crossings showed no significant difference (*p* = .55). Seed set was significantly lower in intra‐ compared with interpopulation crossings (means 65.01% and 77.85%; Chi‐squared = 9.48, *df* = 1, *p* < .01; Table 2, Figure 3b). In contrast, selfings, intra‐ and interlineage crossings (means 42.84%, 32.22%, and 37.42%; Chi‐squared = 1.88, *df* = 2, *p* = .39; Table 2, Figure S1) as well as intra‐ and interpopulation crossings (means 32.66% and 31.14%; Chi‐squared = 0.05, *df* = 1, *p* = .82; Table 2, Figure S2) unveiled comparable germination rates.

**Table 2:** Mean percentages of seed set (percent of viable seeds) and germination rates (germinated seeds) for all lineages and for each lineage according to treatments (selfing, intralineage, and interlineage crossing), and for different intralineage crossing treatments (intra‐ and interpopulation)

Dataset	Treatment	*N_CS_*	*N_S_*	Viable seeds [%]	*N_CG_*	*N_G_*	Germinated seeds (%)
All lineages	Selfing	43	**82**	**2.47^a^**	22	40	42.84^a^
	Intralineage	59	**2,528**	**68.97^b^**	58	871	32.22^a^
	Interlineage	182	**7,228**	**69.49^b^**	182	2,747	37.42^a^
Intralineage	Intrapopulation	**17**	**738**	**65.01^a^**	17	231	32.66^a^
	Interpopulation	**42**	**1,790**	**78.75^b^**	41	640	31.14^a^
lineage 1	Selfing	6	3	0.48^a^	**2**	**3**	**100.00^a^**
lineage 2	Selfing	10	27	5.32^a^	**6**	**15**	**38.38^a^**
lineage 3	Selfing	2	0	0.00^a^	**‐**	**0**	**‐**
lineage 4	Selfing	17	22	1.12^a^	**9**	**5**	**23.15^a^**
lineage 5	Selfing	8	30	3.91^a^	**5**	**17**	**60.79^a^**
lineage 1	Intralineage	10	250	67.44^a^	10	44	16.55^a^
lineage 2	Intralineage	16	539	77.88^a^	16	129	26.07^a^
lineage 3	Intralineage	2	33	50.40^a^	1	21	72.41^a^
lineage 4	Intralineage	22	1,204	66.18^a^	22	376	33.37^a^
lineage 5	Intralineage	9	502	65.77^a^	9	301	53.28^a^
lineage 1	Interlineage	35	**1,257**	**70.90^b^**	35	**261**	**22.03^a^**
lineage 2	Interlineage	57	**2040**	**72.89^b^**	57	**667**	**29.56^ab^**
lineage 3	Interlineage	10	**290**	**67.96^ab^**	10	**150**	**46.70^bc^**
lineage 4	Interlineage	49	**2,611**	**72.98^b^**	49	**1,109**	**42.78^c^**
lineage 5	Interlineage	31	**1,030**	**57.17^a^**	31	**560**	**57.80^c^**

Note

**Figure 4:**
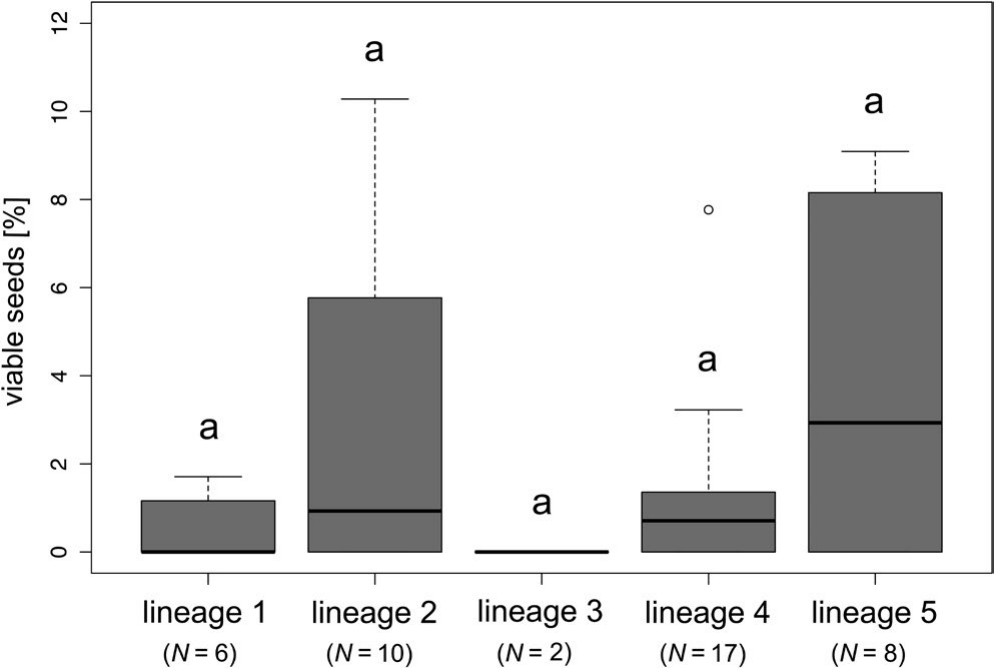
Boxplots showing seed set (percent of viable seeds) of different lineages (selfing treatment). Letters above boxplots indicate nonsignificant differences between groups. *N* = sample size (number of crossings/crossing IDs used to determine seed set)

We marked significant (*p* < .05) Kruskal–Wallis test results in bold. The percentages of viable seeds or germinated seeds per crossing ID (see also Excel file “crossings_2019.xlsx” in Dryad data repository for individuals involved in crossings) were averaged. Superscript letters indicate significant/nonsignificant group differences in post hoc comparisons (e.g., within a dataset, groups with “a” belong together because the post hoc test did not reveal significant differences whereas groups with different letters “a” and “b” are significantly different). See Figures 3, 4, S1–S7 for within‐group variation. The decreased sample size of germination rates is due to crossings with no seed set. *N_CS_* = sample size (number of crossings/crossing IDs used to determine seed set), *N_S_* = number of seeds, *N_CG_* = sample size (number of different crossings/crossing IDs used to determine germination rates), and *N_G_* = number of germinated seeds/seedlings.

Selfings of *R. notabilis* s.l. lineages did not differ significantly from each other in seed set (means 0.00%–5.32%; Chi‐squared = 5.42, *df* = 4, *p* = .25; Figure 4), but in germination rates (means 23.14%–100.00%, Chi‐squared = 8.36, *df* = 3, *p* < .05; Table 2, Figure S3). Nevertheless, we did not detect significant differences in germination rates in pairwise comparisons (*p* > .05), and, in general, investigated differences might be due to the low sample size of lineage 1. Intralineage *R. notabilis* s.l. crossings revealed no significant differences in seed set among lineages (means 50.40%–77.88%; Chi‐squared = 7.75, *df* = 4, *p* = .10; Table 2, Figure S4). Germination rates of intralineage crossings showed significant differences among lineages (means 16.55%–72.41%; Chi‐squared = 12.07, *df* = 4, *p* < .05; Table 2, Figure S5) but also no significant differences in pairwise comparisons (probably due to large within lineage variation). In contrast to intralineage crossings, we observed significant differences in seed set among interlineage ones (means 57.17%–72.89%; Chi‐squared = 19.94, *df* = 4, *p* < .001; Table 2, Figure S6). However, lineage 5 was the only lineage that exhibited significant differences to other lineages (lineages 1, 2, and 4; *p* < .01). Interlineage crossings significantly differed also in relation to germination rates (means 22.03%–57.80%; Chi‐squared = 40.72, *df* = 4, *p* < .001; Table S2, Figure S7). Lineages 1 and 2 (22.03 and 29.56%) showed significantly lower germination rates than lineages 4 and 5 (42.77 and 57.80%; *p* < .05). Lineage 3 (46.70%) differed significantly from lineage 1 (*p* < .05), but not from lineages 2, 4, and 5 (*p* > .05).

Lineage comparisons revealed significantly different genetic distances (means 0.2173–0.3312; Chi‐squared = 111.30, *df* = 14, *p* < .001; Table 3, Figure S8). Individuals within lineages are mostly characterized by the lowest genetic distances (means 0.2173–0.2683) compared to individuals involved in interlineage crossings (means 0.3100–0.3312). Heterozygosity was significantly higher in *R. notabilis* s.l. compared to *R. cassubicifolius* s.l. (means 0.69 versus 0.52, *p* < .05) but not compared to all other diploid sexual species of the *R. auricomus* complex (means 0.52–0.69; Chi‐squared = 9.57, *df* = 3, *p* < .05; Figure S9a). Nevertheless, *R. notabilis* s.l. lineages did not differ with respect to heterozygosity (means 0.54–0.80; Chi‐squared = 6.13, *df* = 4, *p* > .05, Figure S9b).

**Table 3:** Mean genetic distances among diploid *R. notabilis* s.l. lineages based on a general time‐reversible (GTR) model with estimated site frequencies and maximum likelihood (ML)

Lineage	*R. notabilis* s.l. 1	*R. notabilis* s.l. 2	*R. notabilis* s.l. 3	*R. notabilis* s.l. 4	*R. notabilis* s.l. 5
*R. notabilis* s.l. 1	0.2535^a^	0.3110^c^	0.3298^d^	0.3312^d^	0.3293^d^
*R. notabilis* s.l. 2	**–**	0.2173^a^	0.3254^cd^	0.3199^cd^	0.3127^c^
*R. notabilis* s.l. 3	**–**	**–**	0.2683^abcd^	0.3053^bc^	0.3209^cd^
*R. notabilis* s.l. 4	**–**	**–**	**–**	0.2604^a^	0.3100^abc^
*R. notabilis* s.l. 5	**–**	**–**	**–**	**–**	0.2370^abcd^

Superscript letters (a–d) indicate significant/nonsignificant differences between pairs. See also Figure S8 for data distribution.

**Figure 5:**
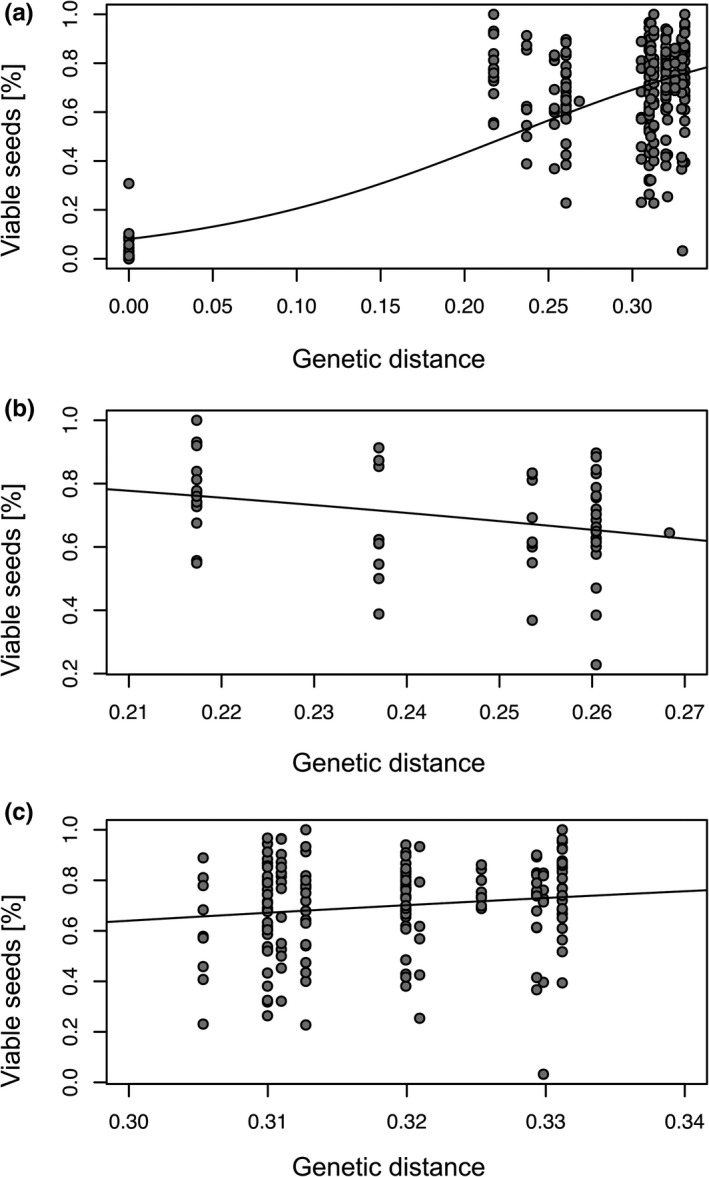
Scatter plots with regression lines based on GLM results of mean seed set (percent of viable seeds) as a function of mean (pairwise) genetic distance. (a) All treatments (selfing, intra‐ and interlineage crossings), (b) intra‐ and (c) interlineage crossings (see Table 3 for pairwise lineage comparisons). The genetic distance of selfings is zero. Regression lines are drawn for significant results (*p* < .05)

**Figure 6:**
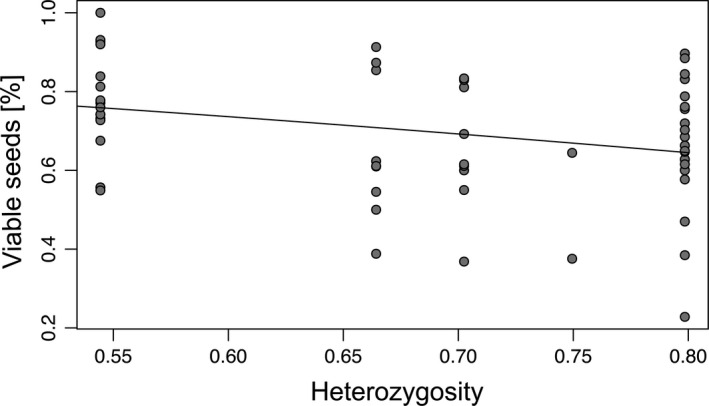
Scatter plot with regression line based on GLM results of mean seed set (percent of viable seeds) as a function of mean heterozygosity of lineages (intralineage treatment)

**Figure 7:**
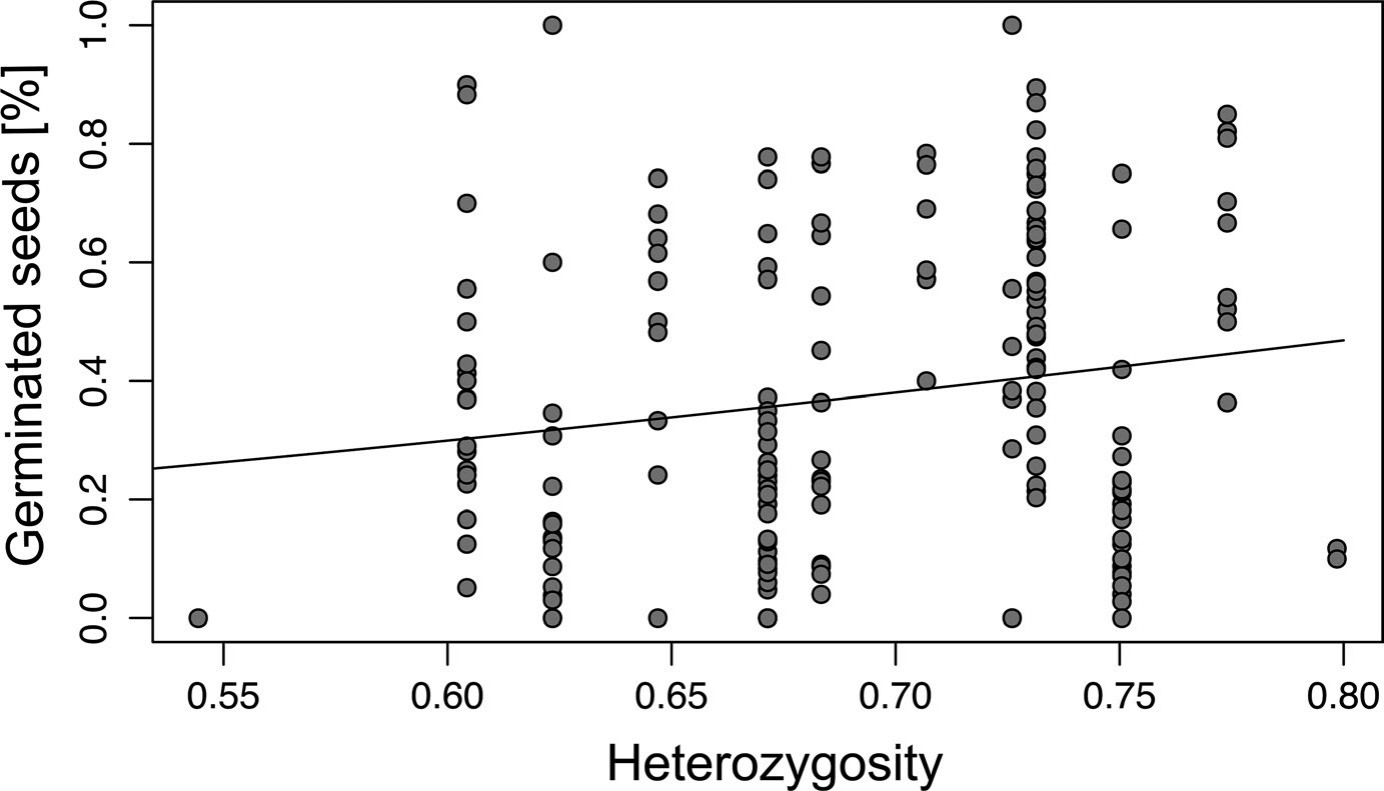
Scatter plot with regression line based on GLM results of mean germination rates (percent of germinated seeds) as a function of mean heterozygosity of lineages (interlineage treatment)

Moreover, we detected significant relationships between seed set and genetic distance (Table 4, Figure 5a–c). In general, seed set and genetic distance were significantly positively related (t = 15.20, *df* = 277/276, *p* < .001; Figure 5a). Whereas the intralineage relationship showed a significantly negative slope (*t* = −2.58, *df* = 58/57, *p* < .05; Figure 5b), the interlineage relationship was also significantly positive (*t* = 2.37, *df* = 175/174, *p* < .05; Figure 5c). Genetic distance did not statistically affect germination rates in general and intra‐ and interlineage (*p* = .51, *p* = .74, and *p* = .38, respectively; Table 4). Intra‐ and interpopulation crossings showed similar relationships (similar slope, though always *p* > .05) between seed set or germination rates and mean genetic distance compared to intralineage crossings (Table 4). In general, and for interlineage crossings, we observed no significant relationships between seed set and mean heterozygosity (*p* = .07 and *p* = .33, respectively; Table 5). In contrast, we found a significant negative relationship between seed set and mean heterozygosity for intralineage crossings (*t* = −2.27, *df* = 58/57, *p* < .05; Table 5, Figure 6). Germination rates were significantly positively related to mean heterozygosity in interlineage crossings (*t* = 2.29, *df* = 181/180, *p* < .05; Figure 7) but not in general and in intralineage crossings (*p* = .21 and *p* = .54, respectively). Intra‐ and interpopulation crossings showed similar relationships (similar slope, though always *p* > .05) between seed set or germination rates and mean heterozygosity compared to intralineage crossings (Table 5). Moreover, we observed a significant isolation‐by‐distance among *R. notabilis* s.l. lineages (*R*_xy_^2^ = 0.22, *p*_xy_ < 0.001; Figure S10).

**Table 4:** GLM results between mean seed set or germination rates for treatments all, intra‐ (intra‐ and interpopulation), and interlineage (response variables) and mean genetic distance (explanatory variable). Degrees of freedom (*df*) are given for null deviance and residual deviance (nd/rd)

Treatment	Mean genetic distance				
	Estimate	Standard error	Degrees of freedom	*t* value	*p* value
Seed set (%)					
All	10.95	0.72	277/276	15.20	***p* < .001**
Intralineage	−13.78	5.33	58/57	−2.58	***p* < .05**
(Intrapopulation)	−10.14	7.69	41/40	−1.32	.19
(Interpopulation)	−4.72	3.57	19/18	−1.32	.20
Interlineage	13.97	5.91	175/174	2.37	***p* < .05**
Germination rates (%)					
All	−0.53	0.81	261/260	−0.66	.51
Intralineage	2.85	8.68	57/56	0.33	.74
(Intrapopulation)	3.73	13.01	40/39	0.29	.78
(Interpopulation)	3.71	7.06	19/18	0.53	.61
Interlineage	−5.62	6.43	181/180	−0.87	.38

**Table 5:** GLM results between mean seed set or germination rates for treatments all, intra‐ (intra‐ and interpopulation), and interlineage crossings (response variables) and mean heterozygosity (explanatory variable). Degrees of freedom (*df*) are given for null deviance and residual deviance (nd/rd)

Treatment	Mean heterozygosity				
	Estimate	Standard error	Degrees of freedom	*t* value	*p* value
Seed set [%]					
All	−1.79	0.99	277/276	−1.82	.07
Intralineage	−2.27	0.96	58/57	−2.27	**<.05**
(Intrapopulation)	−1.72	1.39	41/40	−1.24	.22
(Interpopulation)	−1.34	1.13	19/18	−1.18	.25
Interlineage	−1.19	1.23	175/174	−0.97	.33
Germination rates (%)					
All	1.27	1.02	261/260	1.24	.21
Intralineage	0.96	1.55	57/56	0.62	.54
(Intrapopulation)	1.67	2.34	40/39	0.71	.48
(Interpopulation)	0.58	2.18	19/18	0.27	.79
Interlineage	3.62	1.58	181/180	2.92	**<.05**

## 4 DISCUSSION

In scenarios of geographical parthenogenesis, the astonishing feature is not so much the wide distribution of asexuals, but the restricted distribution of sexual progenitor taxa. The need for mating partners and pollinators for outcrossing, the danger of inbreeding, and a loss of heterozygosity in small populations are potential obstacles for range expansions of sexual species (Baker, 1967; Haag & Ebert, 2004; Hörandl, 2006; Pinc et al., 2020). Here, we analyzed the breeding system of the diploid sexual species *R. notabilis* s.l., a narrowly distributed species within the Illyrian region, and one of the putative progenitors of the Eurasian *R. auricomus* complex. We tested hypotheses whether crossings of adjacent lineages within the species’ range (Karbstein et al., 2020; Tomasello et al., 2020) are self‐incompatible and whether intra‐ and interlineage crossings affect reproductive fitness differentially. We compared reproductive features with levels of genetic distance and genome‐wide heterozygosity in natural *R. notabilis* s.l. lineages.

The test for self‐incompatibility of *R. notabilis* s.l. via bagging experiments confirmed predominant seed formation via outcrossing in the sexual taxa of *R. auricomus* complex as already observed by Hörandl (2008; Table 2). However, the self‐compatibility system in *R. notabilis* is not perfect, as a low amount of seeds were formed also in bagged plants (Table 2; Figures 3, 4). This low degree of self‐fertility in the *R. auricomus* complex is probably due to a multi‐locus gametophytic SI system (GSI) as reported from other congeneric species (Lundqvist, 1990, 1994, 1998), which can result in semi‐self‐compatibility due to dominance effects of loci (Richards, 1997). Rare occurrences of selfed seed were also observed in bagging experiments of the alpine diploid sexual *R. kuepferi* (Cosendai et al., 2013) and in alpine buttercups in Australia (Pickering, 1997). Spontaneous mutations of S‐alleles in the pollen are likely not the reason for self‐compatibility, as we observed selfed seeds in most lineages (except lineage 3 *R. notabilis* s.str.). Finally, pseudo‐self‐compatibility could influence the actual efficiency of SI systems: extreme temperatures and early or late pollinations could reduce the expression of SI proteins, and hence allow for occasional self‐pollen tube growth (de Nettancourt, 2001). Although we are not aware of exposures to extreme temperature or off‐time pollinations in our experiments, we cannot rule them out completely, as plants were kept outside in an experimental garden. Additionally, late gametophytic self‐compatibility was already reported for Ranunculaceae (Gibbs, 2014). Altogether, frequencies of selfed seeds are below 5% (with outliers up to ca. 15%–31%), which makes it questionable that selfing played a major role in range expansion scenarios. In past glacial times, selfing rates could have been potentially higher due to cold‐induced pseudo‐self‐compatibility, but climate chamber experiments with cold stress treatments would have to verify this assumption. Eventually, occasional selfing helped to disperse lineages within the species range, but long‐distance dispersal appears to play no major role for *R. notabilis* s.l.. In the *R. auricomus* complex, achenes are mainly dispersed by ants, but potentially also by wind (due to a cavity between achene and seed; Müller‐Schneider, 1986). Biogeographical analysis of sexual species of the complex, however, did not support a hypothesis of long‐distance dispersal but suggested overall a vicariance scenario (Tomasello et al., 2020). In contrast, polyploid apomicts of the *R. auricomus* complex are fully self‐fertile, which appears to be a major advantage for range expansions in GP scenarios (Kirchheimer et al., 2018) and also for long‐distance dispersal (Cosendai & Hörandl, 2010). Polyploid apomicts of the *R. auricomus* complex indeed occur in isolated, remote areas (e.g., on Iceland, Svalbard, but also in Southern Europe, see maps in Hörandl, 2009), which have more likely been reached via long‐distance dispersal.

The inter‐ and intralineage crossings in *R. notabilis* s.l. revealed no significant differences in seed set (Table 2, Figure 3a). Hence, full crossability is confirmed within *R. notabilis* s.l., which fits the observation of a shared gene pool, very low genetic distances, and a similar morphology of lineages, justifying the taxonomic treatment as a single species (Karbstein et al., 2020). Apparently, no crossing barriers exist within *R. notabilis* s.l., and adjacent lineages have a potential for gene flow. The variation of seed set (Figures 3, 4, S4, S6) is similar to results for other sexual species of the *R. auricomus* complex (ca. 60% in Hörandl, 2008; 78% in Hojsgaard et al., 2014). In contrast, interspecific crossings between the genetically more diverged diploid species *R. notabilis* s.str. and *R. cassubicifolius* s.l. revealed a drastically reduced seed set (10%–20% viable seeds), disturbances of meiosis and sporogenesis, and shifts to apomixis (Barke et al., 2018; Hojsgaard et al., 2014; Hörandl, 2008). Cross‐pollinations in nonemasculated flowers eventually can induce spontaneous selfing, when foreign pollen originated from a different species, from another cytotype, or was also partly aborted (Mentor effects; de Nettancourt, 2001; Hörandl and Temsch, 2009). However, none of these conditions applied to our crossings, as all lineages are diploid, conspecific, and do have excellent pollen quality (Dunkel et al., 2018; Hörandl et al., 1997). Mentor effects occurred in interploidal crossings of *R. auricomus* cytotypes at very low frequencies (1.3% of all seeds; Hörandl and Temsch, 2009). Therefore, we regard selfed seeds resulting from Mentor effects as unlikely and negligible in our crossing experiments on *R. notabilis* s.l..

Seed set is apparently the main decisive factor for female fitness as germination rates do not differ between selfed, intra‐, or interlineage crossings. This observation and the range of variation of germination rates among treatments and lineages (Figs S1–3, S5, S7) are also in accordance with previous observations in the *R. auricomus* complex (Barke et al., 2018; Hörandl, 2008; Lohwasser, 2001). Hence, we regard the differences in germination rates among *R. notabilis* lineages not necessarily as lineage‐specific. Pollen quality is for all lineages of *R. notabilis* s.l. high with more than ca. 90% good pollen (Hörandl et al., 1997; Dunkel et al., 2018), and hence effects of male fitness on seed set are probably negligible.

We measured genetic differentiation and genome‐wide heterozygosity in natural populations to understand their effects on breeding systems. Genomic markers like RADseq loci provide a genome‐wide estimate of genetic differentiation and heterozygosity, and hence can be informative also with a low number of sampled individuals (Lovell et al., 2014; Park & Donoghue, 2019). In general, we found a positive relationship between seed set and genetic distance (Table 4, Figure 5a): the more genetically distant the individuals involved in crossings, the higher the seed set. However, different mechanisms are probably responsible for the reduction of seed set when genetically identical or similar pollen is involved: either the GSI system inhibiting self‐pollination, or inbreeding depression resulting from crossings of genetically similar individuals (see e.g., intrapopulation crossings). This suggests a positive effect of outbreeding, probably due to increased genetic diversity and decreased inbreeding effects in the offspring (Gai & Lu, 2013; Wirth et al., 2011). Interestingly, we observed positive relationships between interlineage seed set and mean genetic distance, and interlineage germination rates and mean heterozygosity (Tables 4, 5, Figures 5c, 7). Sufficient genetic distance and heterozygosity among crossed interlineage individuals might prevent seed abortion due to lethal alleles and might ensure embryo and endosperm development, and therefore allow better seed set and germination rates. Instead of inbreeding depression, the multi‐locus SI system can also partly explain the decreased seed set. Gametophytic SI systems rely on a high diversity of S‐alleles within a population (Richards, 1997), which might be eroded in small, genetically depauperated populations. We have no direct measures of S‐allele diversity in our lineages. However, our genotypes of different lineages were genetically more distant than genotypes within lineages (Table 3, Figure S8), suggesting sufficient allelic diversity in interlineage crossings. Thus, we consider inbreeding depression as a more reasonable explanation. Inbreeding effects may partly explain the remarkably lower seed set of intra‐ compared to interpopulation crossings (Figure 3b). Outbred progeny may benefit from heterosis effects, potentially explaining the higher germination rates generated by crossings of more genetically diverse interlineage individuals. In diploid sexuals of the *Hieracium alpinum* complex also characterized by a GP pattern, the inbred progeny showed lower biomass than the outbred progeny, probably contributing to lower colonizing ability of sexuals compared with apomictic conspecifics (Pinc et al., 2020).

Within our intralineage crossings, in contrast, seed set was decreased, when genetically more distant individuals or individuals with high heterozygosity were crossed (Tables 3, 5, Figures 5b, 6). We observed similar relationships between intra‐ und interpopulation crossings compared with intralineage crossings in total, indicating that probably similar processes act on these relationships. An explanation might be outbreeding depression by crossings of different individuals within small populations that was already demonstrated and is probably related to adaptation to environmental variation (microhabitats) or the coadaptation of genes at different loci (e.g., *Anchusa crispa*; Quilichini et al., 2001). The absence of inbreeding depression would have been more reasonable in relation to the other results. However, we observed a reduced seed set in intra‐ compared with interpopulation crossings, ruling out the explanation of absent inbreeding depression. Different processes may act on intra‐ and interlineage crossings. However, we could not detect a general positive effect of increased heterozygosity on seed set and germination rates probably due to sufficient genetic variation within and only minor genetic differences among parental *R. notabilis* s.l. individuals.

In a microevolutionary context, we suppose that interlineage crossings have repeatedly reshuffled the gene pool of the species, and avoided strong inbreeding in small populations. Gene flow could have happened during the evolutionary history of the species in the last cold periods (see Tomasello et al., 2020). In phylogenetic reconstructions of the *R. auricomus* complex, incongruence and signals for introgression were found among lineages of *R. notabilis* s.l., and these lineages showed also a shared gene pool in STRUCTURE analyses (Karbstein et al., 2020). Gene flow might be expected in extant sympatric populations in Slovenia (Dunkel et al., 2018). However, the partial self‐compatibility of *R. notabilis* potentially explains beside isolation‐by‐distance (Figure S10) the slight genetic differentiation among lineages despite their partial sympatric occurrence. Prezygotic barriers, for example, differences in flowering time (nonoverlapping flowering periods), were not apparent among diploid sexual *R. notabilis* s.l. lineages from the collection dates of Dunkel et al. (2018) and Hörandl and Gutermann (1998), and lineages flowered synchronously at the beginning of our crossing experiments. Individuals of the *R. auricomus* complex form cup‐shaped generalist flowers (Steinbach & Gottsberger, 1995), which makes pollinator specificity also unlikely. A population genetic study with a more dense sampling over the whole area, however, is needed to settle the actual amount of gene flow within and between lineages. In other cases of GP, geographically more isolated sexual species showed a much more pronounced geographical population structure and genetic differentiation, for example, in *R. kuepferi* in the Alps and *Crataegus* in North America (Cosendai et al., 2013; Lo et al., 2009). Genome‐wide heterozygosity levels as measured here with RADseq data within *R. notabilis* s.l. are comparable to other species of the complex and even higher than in the widespread species *R. cassubicifolius* s.l. (Figure S9a), another progenitor of the *R. auricomus* complex. The latter species has a strongly disjunct distribution around the Northern and Southern Alps and in the Carpathians, with a much stronger geographical isolation between lineages (see Tomasello et al., 2020), which may reduce heterozygosity and genetic diversity due to inbreeding.

We observed a decreased female fitness in intrapopulation crossings and in crossings between genetically similar or less heterozygous individuals of different lineages (Tables 2, 4, 5, Figures 3b, 5c, 7). Self‐incompatibility and the tendency of inbreeding depression in small and/or isolated populations are probably partly responsible for the limited geographic ranges of sexual lineages of *R. notabilis* s.l.. However, here, the outcrossing system appears to be fully functional, and besides the reduced seed set in intrapopulation crossings, no signs of strong inbreeding are apparent from our data. Hence, it is plausible that the species can maintain and might even expand its distributional range and would not be at risk of extinction within the Illyrian region. Population genetic studies on the most isolated lineage of *R. notabilis* s.str. in Austria showed a population structure typical for outcrossers, with a tendency to inbreeding in one population (Hörandl et al., 2000). Natural forest habitats in this area are partly destroyed by spruce forestation, which results in acidification of soils and devastation of the understory flora (pers. obs. of authors). However, *R. notabilis* s.l. also occurs on humid nutrient‐poor anthropogenic meadows that are, despite recent nature conservation efforts, still under pressure due to habitat eutrophication, draining, and defragmentation. These recent factors probably limit not only range expansion, but contribute to fragmentation of the distribution area. In deeper time levels, that is, in the postglacial reforestation scenarios of Central Europe, apomictic polyploid congeners might have colonized faster newly available habitats, as shown by Kirchheimer et al. (2018) for *R. kuepferi*. However, also the sympatric emergence of apomicts can play a major role in GP scenarios (Nardi et al., 2020). Biotic interactions of diploids of the *R. auricomus* complex with polyploid apomictic conspecifics or crossings with sympatric tetraploid apomicts as pollen donors may result in introgression and loss of fertility in triploid offspring (see Hörandl et al., 2000), potentially also reducing fitness of *R. notabilis* s.l. populations. Hence, in mixed populations, diploid outcrossing sexuals would suffer from a minority cytotype disadvantage (Levin, 1975) while polyploid self‐fertile apomicts would not be fertilized and hence not affected.

## 5 CONCLUSION

We confirm predominant self‐incompatibility of sexual *R. notabilis* s.l., as a general disadvantage for rapid colonization and range expansions, as predicted by Baker's law. In GP scenarios, small and disjunct distribution areas of sexual species in relic areas confer the potential disadvantage of inbreeding of genetically similar individuals. We confirmed in our model system a reduced female fitness in intrapopulation (a part of intralineage) crossings. We also found higher female fitness (seed set) with increasing genetic distance in all and interlineage crossings, and higher germination rates with increasing heterozygosity in interlineage crossings. These results support an interpretation that inbreeding depression particularly affects intrapopulation (intralineage) crossings, and that positive outbreeding effects particularly influence interlineage crossings. Breeding systems and genetic properties of sexual species are important, although not the exclusive factor for the restricted distribution ranges of sexual species and have to be considered for understanding GP patterns.

## CONFLICT OF INTEREST

None declared.

## AUTHOR CONTRIBUTIONS

**Kevin Karbstein:** Conceptualization (supporting); Formal analysis (lead); Investigation (equal); Methodology (lead); Visualization (lead); Writing‐original draft (lead); Writing‐review & editing (lead). **Elisabeth Rahmsdorf:** Conceptualization (supporting); Formal analysis (supporting); Investigation (lead); Methodology (supporting); Visualization (supporting); Writing‐original draft (equal); Writing‐review & editing (supporting). **Salvatore Tomasello:** Conceptualization (supporting); Investigation (supporting); Supervision (supporting); Writing‐original draft (supporting); Writing‐review & editing (supporting). **Ladislav Hodač:** Investigation (supporting); Resources (supporting); Writing‐original draft (supporting); Writing‐review & editing (supporting). **Elvira Hörandl:** Conceptualization (lead); Formal analysis (supporting); Funding acquisition (lead); Methodology (equal); Project administration (lead); Resources (supporting); Supervision (lead); Visualization (supporting); Writing‐original draft (lead); Writing‐review & editing (lead).

## Supporting information

Fig S1‐S10

## Data Availability

Code availability. The R script used in data analyses are deposited on Dryad (https://doi.org/10.5061/dryad.66t1g1k10). Basic data supporting the findings of this study are available within the manuscript and the Appendix. Counts and statistics of crossing and germination experiments are deposited on Dryad (https://doi.org/10.5061/dryad.66t1g1k10). Demultiplexed RADseq reads are stored on National Center for Biotechnology Information Sequence Read Archive (SRA): BioProject ID PRJNA627796.
