# Supplementary material for: Breeding system of diploid sexuals within the Ranunculus auricomus complex and its role in a geographical parthenogenesis scenario
Source: Ecol Evol. 2020 Dec 3;10(24):14435–50. doi: 10.1002/ece3.7073 (PMC7771175; doi:10.1002/ece3.7073)
Supplement: Supplementary file 1 — Fig S1‐S10 [file ECE3-10-14435-s001.docx]

**Breeding system of diploid sexuals within the *Ranunculus auricomus* complex and its role in a geographical parthenogenesis scenario**

**Karbstein, Kevin^1,2^, Rahmsdorf, Elisabeth^1,#^, Tomasello, Salvatore^1^, Hoda****č, Ladislav^1^ & Hörandl, Elvira^1,*^**

**1** University of Göttingen, Albrecht-von-Haller Institute for Plant Sciences, Department of Systematics, Biodiversity and Evolution of Plants (with Herbarium), Untere Karspüle 2, D-37073, Göttingen, Germany

**2** University of Göttingen, Georg-August University School of Science (GAUSS), Wilhelmsplatz 1, D-37073, Göttingen, Germany

^#^ current address: Leipzig University, Institute of Biology, Johannisallee 21-23, D-04103, Leipzig, Germany

*** contact address corresponding author:** elvira.hoerandl@biologie.uni-goettingen.de

**
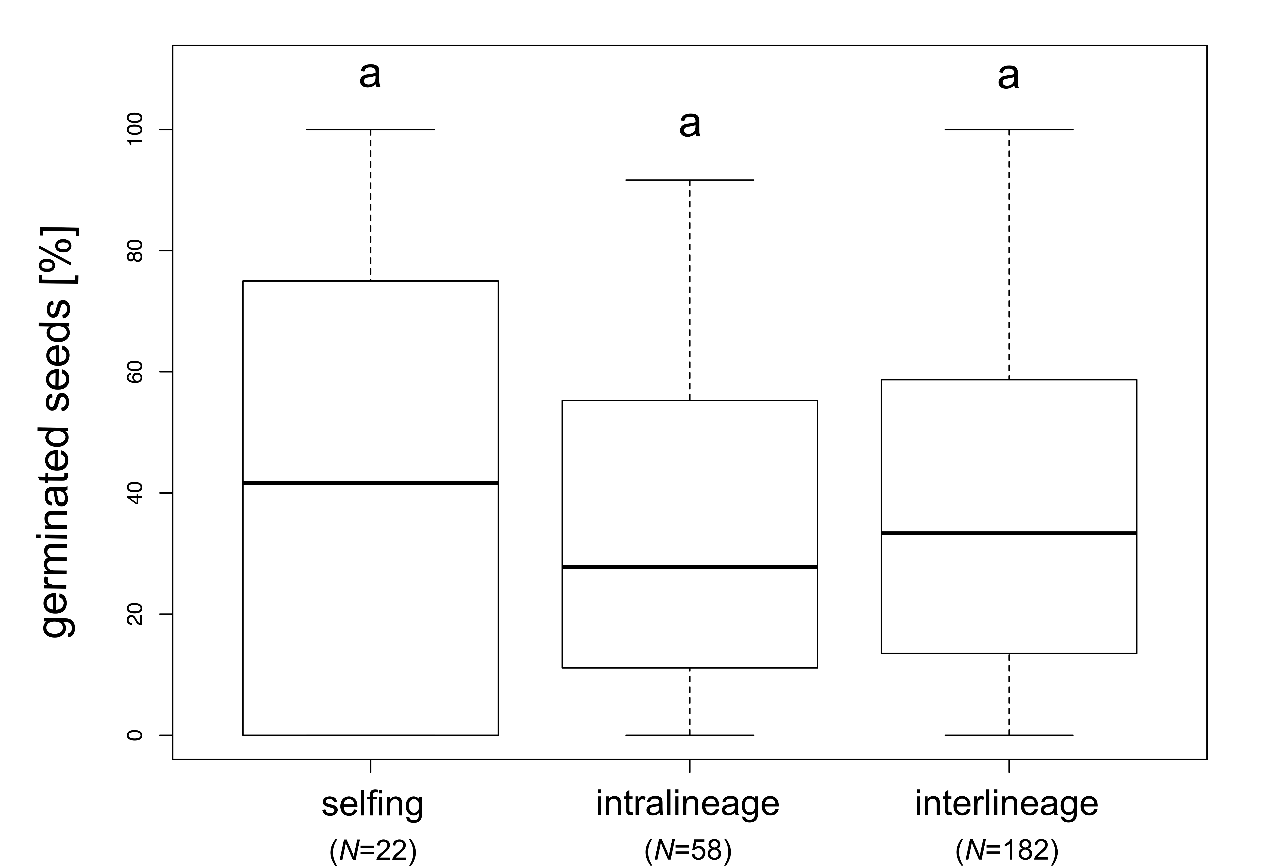
**

**Fig. S1.** Boxplots showing germination rates of different treatments (selfing, intra-, and interlineage). Germination rate = percent of germinated seeds in relation to viable seeds. Letters above boxplots indicate non-significant differences between groups. See Table 2 for details. *N* = sample size (number of different crossings/crossing IDs used to determine germination rates).


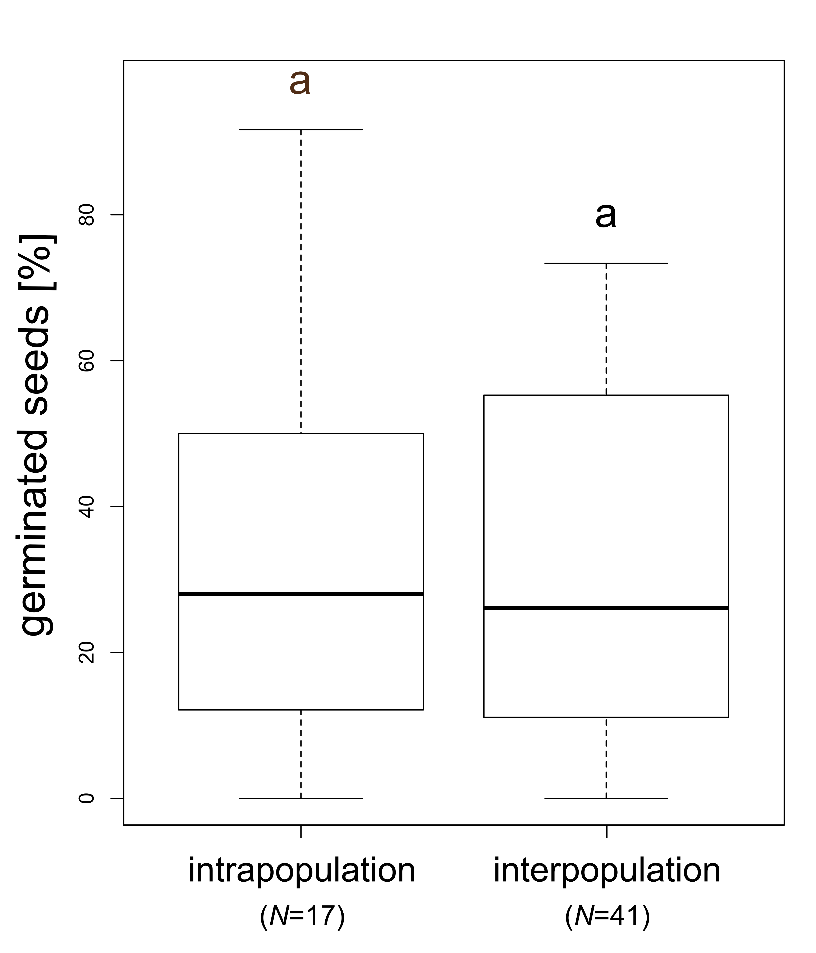


**Fig. S2.** Boxplots showing germination rates of intra- und interpopulation crossings (see also Fig. 3b for seed set of intra- and interpopulation crossings). Germination rate = percent of germinated seeds in relation to viable seeds. Letters above boxplots indicate non-significant differences between groups. See Table 2 for details. *N* = sample size (number of different crossings/crossing IDs used to determine germination rates).


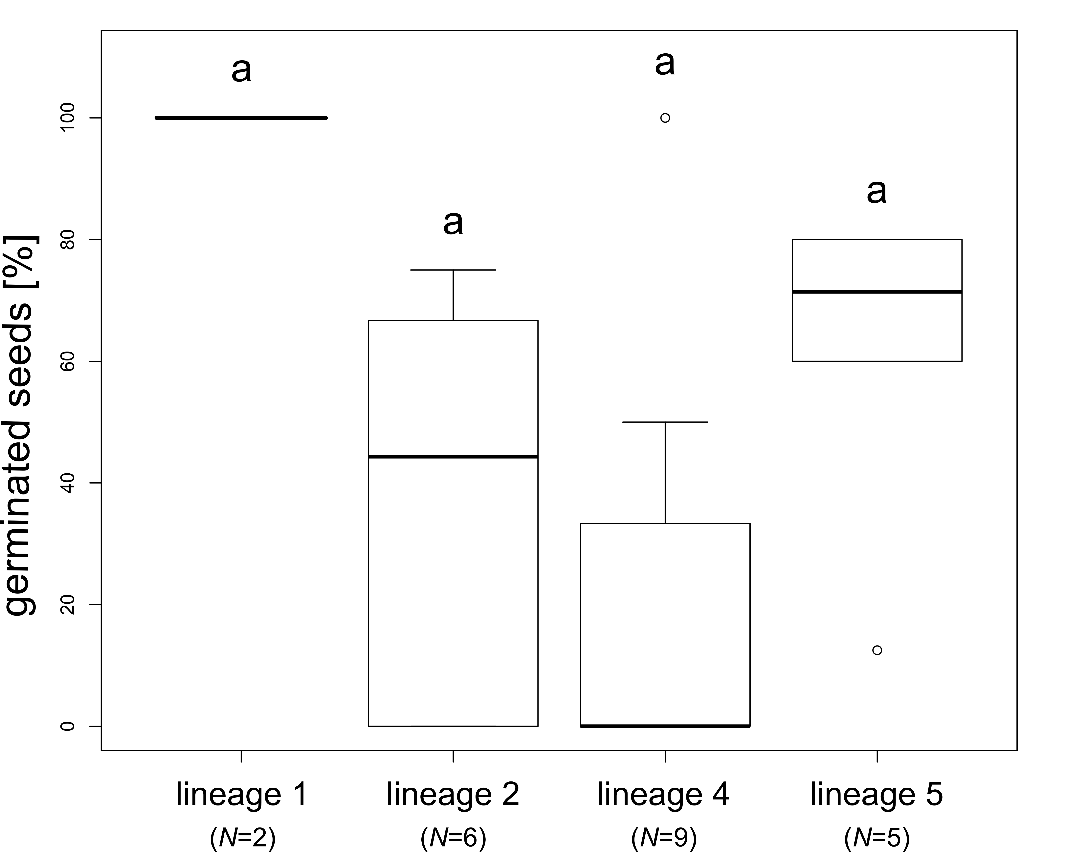


**Fig. S3.** Boxplots showing germination rates of different lineages (selfings). Germination rate = percent of germinated seeds in relation to viable seeds. Letters above boxplots indicate non-significant differences between groups. See Table 2 for details. *N* = sample size (number of different crossings/crossing IDs used to determine germination rates).


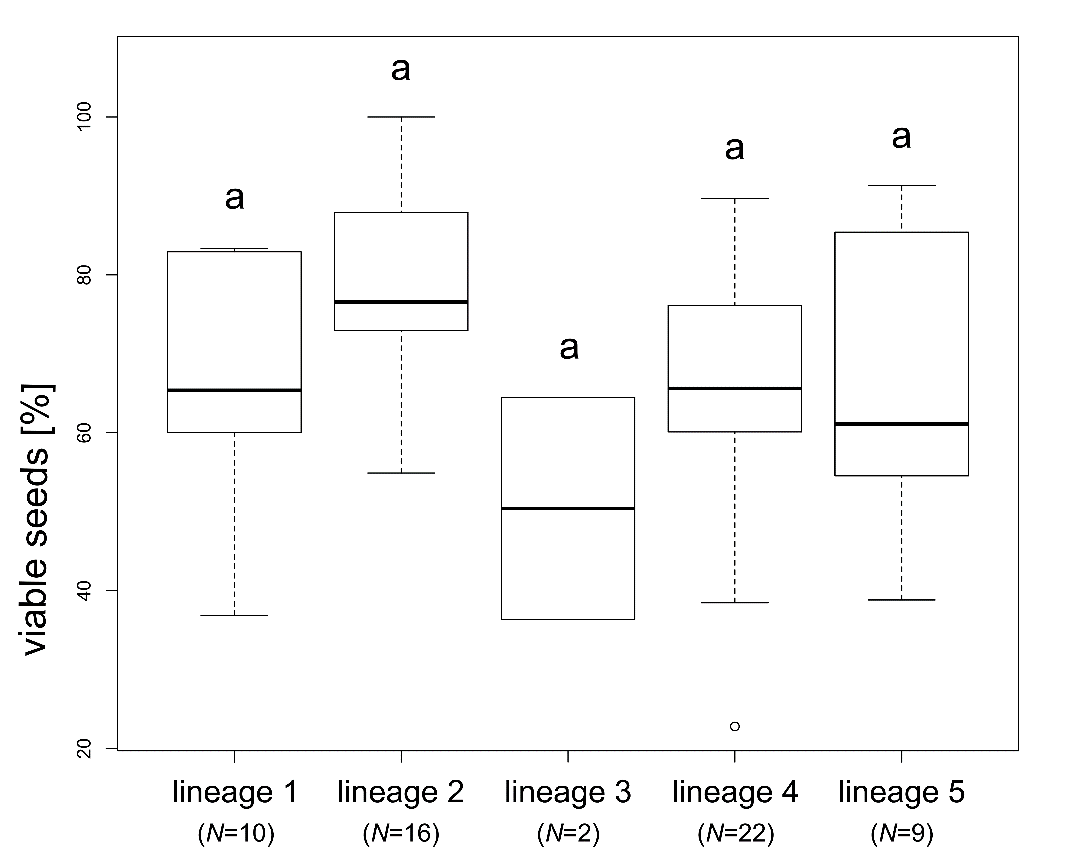


**Fig. S4.** Boxplots showing seed set of different lineages (intralineage crossings). Seed set = percent of viable seeds in relation to all seeds. Letters above boxplots indicate non-significant differences between groups. See Table 2 for details. *N* = sample size (number of crossings/crossing IDs used to determine seed set).


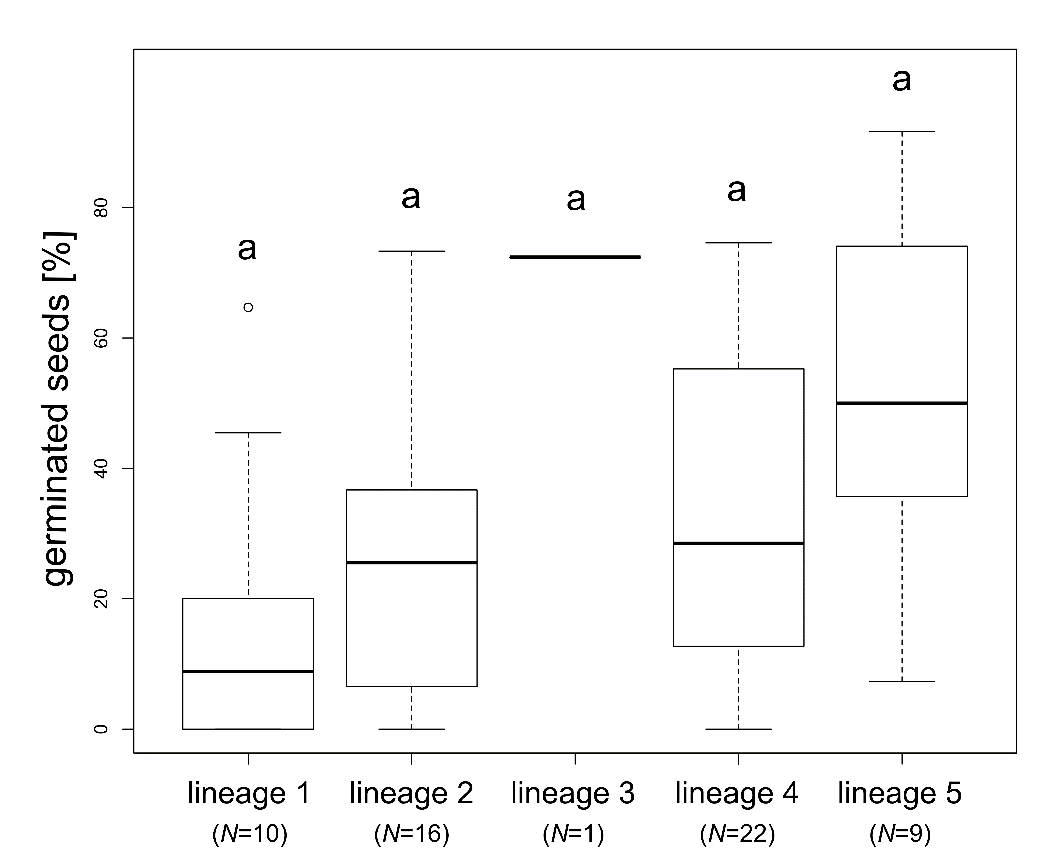


**Fig. S5.** Boxplots showing germination rates of different lineages (intralineage crossings). Germination rate = percent of germinated seeds in relation to viable seeds. Letters above boxplots indicate non-significant differences between groups. See Table 2 for details. *N* = sample size (number of different crossings/crossing IDs used to determine germination rates).


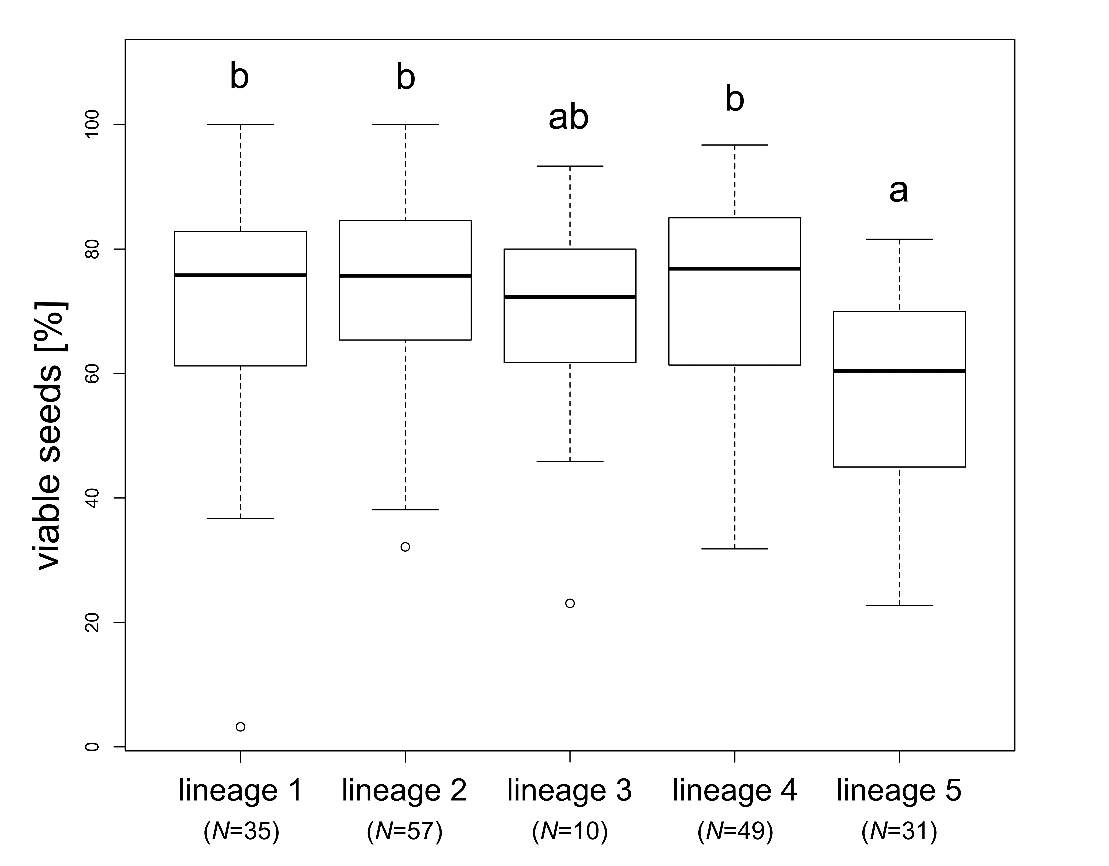


**Fig. S6.** Boxplots showing seed set of different lineages (interlineage crossings). Seed set = percent of viable seeds in relation to all seeds. Letters above boxplots indicate significant/non-significant differences between groups. See Table 2 for details. *N* = sample size (number of crossings/crossing IDs used to determine seed set).


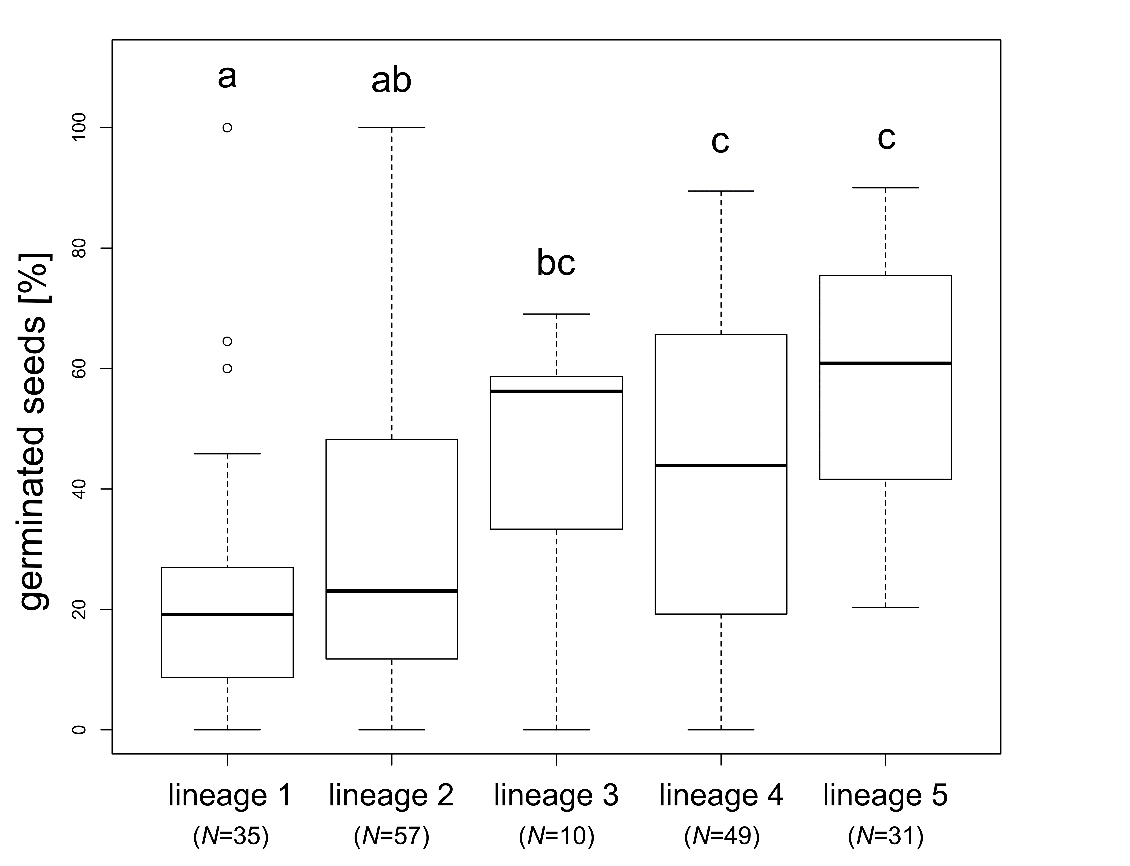


**Fig. S7.** Boxplots showing germination rates of different lineages (interlineage crossings). Germination rate = percent of germinated seeds in relation to viable seeds. Letters above boxplots indicate significant/non-significant differences between groups. See Table 2 for details. *N* = sample size (number of different crossings/crossing IDs used to determine germination rates).

**
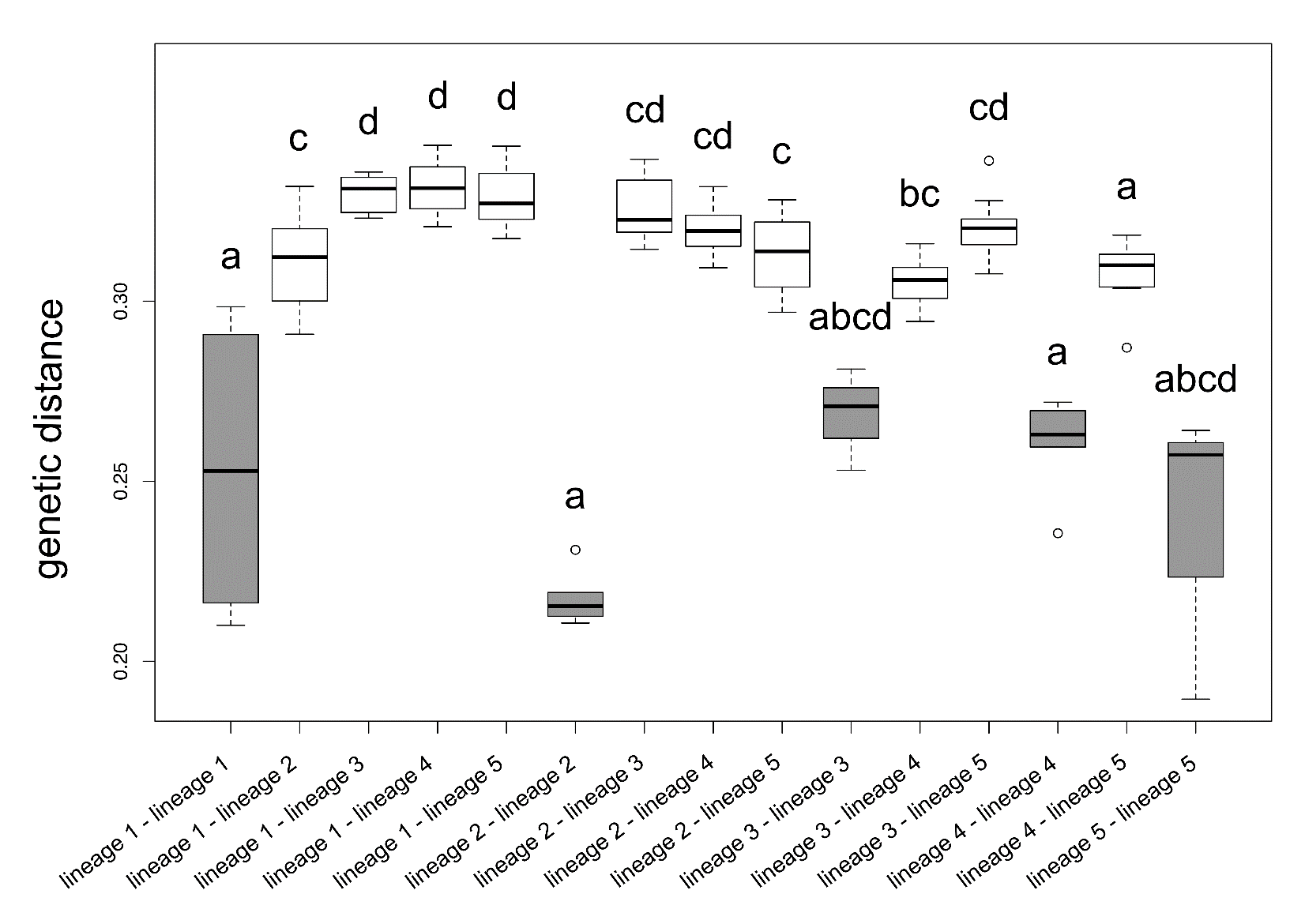
**

**Fig. S8.** Boxplots showing genetic distances of different lineage combinations (intra- (e.g., ‘lineage 1 - lineage 1’; boxplots with grey filling) and interlineage (e.g., ‘lineage 2 - lineage 4; boxplots with white filling)); lineages indicated by numbers). Letters above boxplots indicate significant/non-significant differences between groups. See Tables 1, 3 for details.


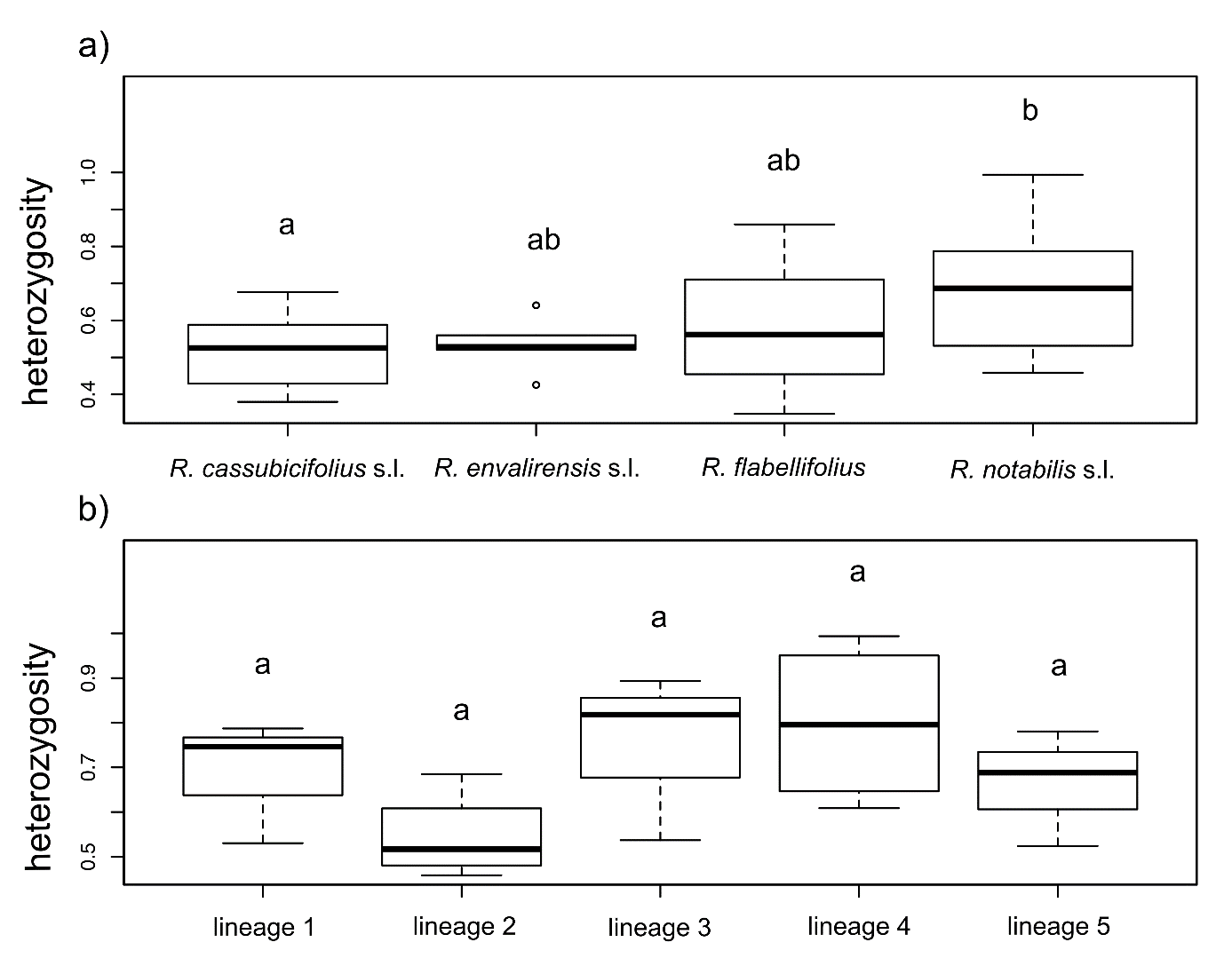


**Fig. S9.** Boxplots showing (a) individual heterozygosity values among four sexual species of the *R. auricomus* complex and (b) individual heterozygosity values among *R. notabilis* lineages (data from Karbstein et al., subm.). Letters above boxplots indicate significant/non-significant differences between groups.


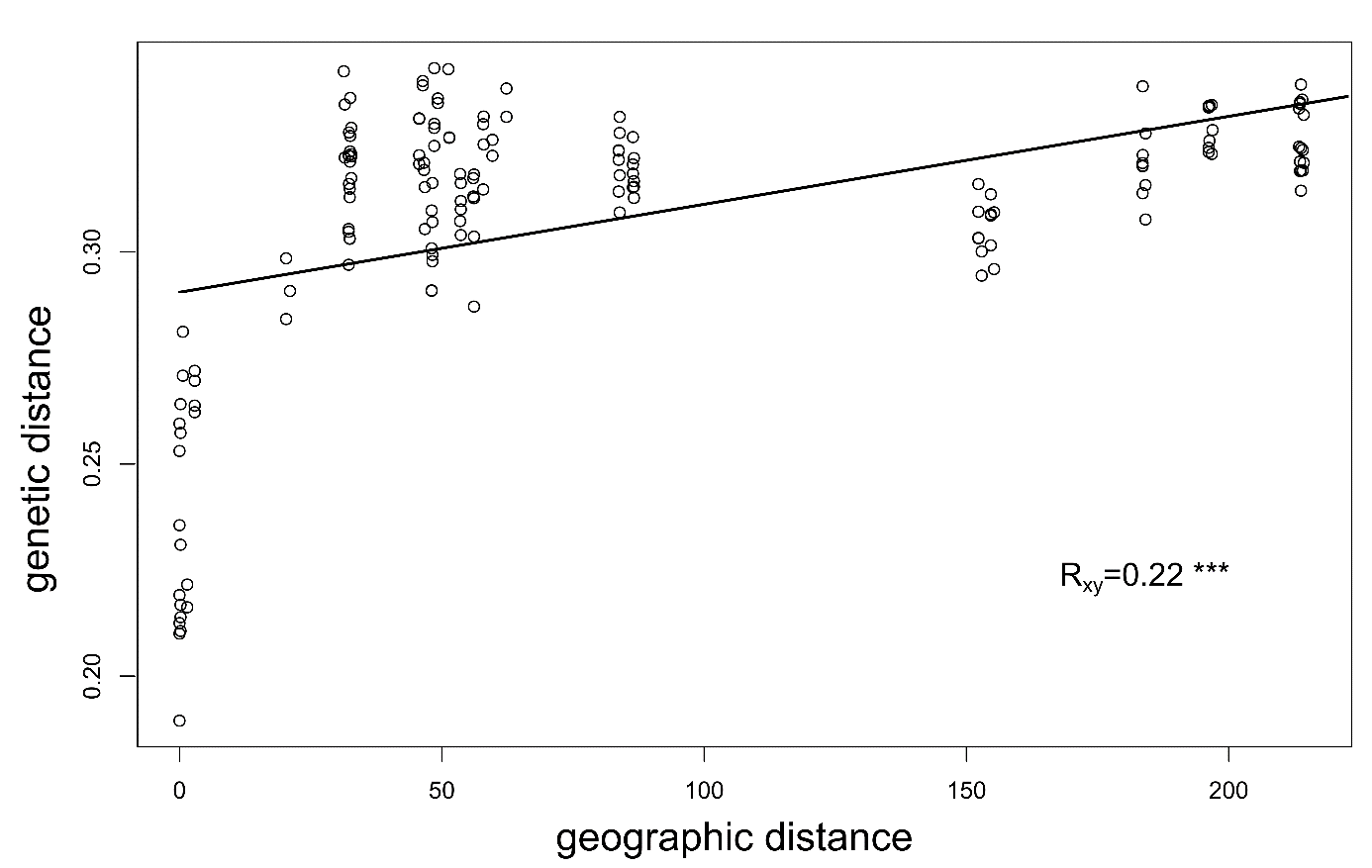


**Fig. S10.** Scatter plot with a regression line based on Mantel test results (Spearman; 9999 permutations) of genetic distance as a function of geographical distances among individuals of *R. notabilis* s.l. lineages.
